# Endoplasmic Reticulum Stress Activates Unfolded Protein Response Signaling and Mediates Inflammation, Obesity, and Cardiac Dysfunction: Therapeutic and Molecular Approach

**DOI:** 10.3389/fphar.2019.00977

**Published:** 2019-09-10

**Authors:** Omar Mohammed Amen, Satyajit D. Sarker, Reena Ghildyal, Aditya Arya

**Affiliations:** ^1^School of Bioscience, Faculty of Health and Medical Sciences, Taylor’s University, Subang Jaya, Malaysia; ^2^Centre for Natural Products Discovery, School of Pharmacy and Biomolecular Sciences, Liverpool John Moores University, Liverpool, United Kingdom; ^3^Centre for Research in Therapeutic Solutions, Faculty of Science and Technology, University of Canberra, Canberra, Australia; ^4^Department of Pharmacology and Therapeutics, Faculty of Medicine, Dentistry and Health Sciences, University of Melbourne, Parkville, VIC, Australia; ^5^Department of Pharmacology and Therapeutics, School of Medicine, Faculty of Health and Medical Sciences, Taylor’s University, Subang Jaya, Malaysia; ^6^Malaysian Institute of Pharmaceuticals and Nutraceuticals, Bukit Gambir, Malaysia

**Keywords:** obesity, cardiac dysfunction, endoplasmic reticulum stress, unfolded protein response, inflammation, insulin resistance, oxidative stress

## Abstract

Obesity has been implicated as a risk factor for insulin resistance and cardiovascular diseases (CVDs). Although the association between obesity and CVD is a well-established phenomenon, the precise mechanisms remain incompletely understood. This has led to a relative paucity of therapeutic measures for the prevention and treatment of CVD and associated metabolic disorders. Recent studies have shed light on the pivotal role of prolonged endoplasmic reticulum stress (ERS)-initiated activation of the unfolded protein response (UPR), the ensuing chronic low-grade inflammation, and altered insulin signaling in promoting obesity-compromised cardiovascular system (CVS). In this aspect, potential ways of attenuating ERS-initiated UPR signaling seem a promising avenue for therapeutic interventions. We review intersecting role of obesity-induced ERS, chronic inflammation, insulin resistance, and oxidative stress in the discovery of targeted therapy. Moreover, this review highlights the current progress and strategies on therapeutics being explored in preclinical and clinical research to modulate ERS and UPR signaling.

## Introduction

Obesity is a major health problem worldwide and often associated with an increased prevalence of cardiovascular diseases (CVDs) and premature death. According to the World Health Organization (WHO), there were over 1.9 billion overweight adults in 2016, of whom 650 million were obese (WHO, 2018). There are also 2.6 million estimated annual deaths associated with obesity-related diseases.

An increase in the prevalence of obesity entails an increase in confounding cardiometabolic disorders. Some of these obesity-linked cardiometabolic health problems include CVD, type 2 diabetes mellitus (T2DM), hypertension, myocardial infarction, atherosclerosis, and stroke (Poirier et al., 2006). According to WHO (2017), an estimated 17.7 million people died from CVD in 2015, of which 13% were because of coronary heart disease. Epigenetics and traditional risk factors such as hypercholesterolemia, hyperlipidemia, T2DM, and hypertension are significant contributors to the increasing prevalence of CVD. However, obesity is considered an independent factor for the increased prevalence of different subtypes of CVD including hypertension, stroke, coronary heart disease (CHD), heart failure (HF), and atrial fibrillation (AF) (Lavie et al., 2014; Ndumele et al., 2016).

Currently, various preventive public health strategies ranging from managing the risk factors, lifestyle change, physical exercise, and smoking cessation aimed at reducing the incidence of CVD are in implementation (Tomé-Carneiro and Visioli, 2016). Several pharmacological drugs, such as propranolol, digoxin, hydrochlorothiazide, procainamide, atorvastatin, lovastatin, rosuvastatin, simvastatin, beta-blockers, and angiotensin-converting enzyme (ACE) inhibitors are also in use for management of CVDs (Opie, 2000). Although the number of therapeutic agents used for the management of cardiovascular pathologies has increased steadily, there is also growing concern over serious and frequent adverse drug reactions induced by commonly used cardiovascular drugs. The most common drug effects induced by cardiovascular drugs are toxicity, anorexia, nausea, vomiting, bradycardia, headache, weakness, hypokalemia, and hyperuricemia (Tomé-Carneiro and Visioli, 2016). According to an observational, prospective, case–series study conducted in Iran, the rate of adverse drug reactions induced by cardiovascular drugs was 24.2%. The most frequently vulnerable sites for cardiovascular drug adverse effects were the central/peripheral nervous systems and the gastrointestinal systems, accounting for 23.5% and 16.5%, respectively (Mohebbi et al., 2010). In addition, some of these drugs were reported with pharmacokinetic or pharmacodynamic interactions, most prevalently on the muscular system, hepatic function, and renal function, leading to interference with the metabolism or hemodynamic of another drug, which is used as co-treatment (Opie, 2000; Anderson and Nawarskas, 2001). Entirely, this has resulted in apparent limitations in effective therapeutics for CVD. Therefore, the search for better treatment strategies and options to overcome these challenges remains a priority.

Knowledge of underlying mechanisms interrelating obesity and cardiac dysfunction is essential to identify potential therapeutic targets for cardiovascular dysfunctions. In the last few decades, significant effort has been made to elucidate the association between obesity and CVD. However, the biomolecular events and mechanisms governing the development of obesity-related CVD remain unclear. This has led to a relative paucity of therapeutic measures for the prevention and treatment of obesity-related CVD and associated metabolic disorders.

Interestingly, numerous *in vitro* and animal studies have documented the role of endoplasmic reticulum stress (ERS) in the pathophysiology of different subtypes of CVD. Recent studies have highlighted the pivotal role of prolonged ERS-initiated activation of the unfolded protein response (UPR), the ensuing chronic low-grade inflammation, and altered insulin signaling in promoting obesity-compromised cardiovascular system (CVS) (Hotamisligil, 2010; Thorp, 2012; Liu and Dudley, 2014; Szymański et al., 2017; Zhang et al., 2017b). However, the precise mechanism of these phenomena is not clearly understood. Therefore, a thorough understanding of how obesity causes persistent ERS in the myocardium and triggers provocative activation of the UPR signaling, inflammation, and insulin resistance is critical.

In the current article, we review the existing knowledge of obesity-linked CVD, ERS, inflammation, insulin resistance, and oxidative stress in the pathophysiology of obese compromised CVS. Moreover, we summarize molecular mechanisms of ERS-associated CVDs, highlighting common interplaying mechanisms that could be of tendency for future novel drug discovery. Furthermore, our review will discuss current progress in CVD therapeutics and the roles of phytochemicals and small molecules as potential therapeutics for the prevention and treatment of CVDs, emphasizing new directions for studying the pathophysiology of CVDs and for developing novel drugs targeting ERS and UPR.

## Correlation Between Obesity and Cardiac Dysfunction

Obesity is a nutritional disorder that results from an accumulation of body fat in certain parts of the body in a way that adversely affects health. It often occurs due to an imbalance between dietary intake and energy expenditure. The association between obesity and CVD is a well-established phenomenon. Data from experimental, epidemiological, and clinical studies, including the Framingham Heart Study, the Manitoba Study, and the Harvard Public Health Nurses Study (Poirier et al., 2006; Van Gaal et al., 2006), support the notion that obesity is an independent risk factor and leads to adverse cardiometabolic effects. Of significant note, in the Framingham Heart Study involving the echocardiography (ECG) of healthy and obese individuals, the latter had increased left ventricular hypertrophy (Siti et al., 2015). Moreover, several experimental and clinical studies have shown the role of excess calories, an underlying problem leading to obesity, in the development of obesity-associated CVD, including cardiac hypertrophy and myocardial dysfunction (Guo et al., 2013). The correlation between excess nutrition/overweight/obesity and other risk factors such as hyperlipidemia, high free fatty acids (FFAs), reactive oxygen species (ROS), ERS, inflammation, and insulin resistance and cardiac dysfunction is illustrated in the schematic diagram in **Supplemental Material**.

## Role of Endoplasmic Reticulum Stress (ERS) in CVD

### Endoplasmic Reticulum (ER)

ER was first discovered by Porter et al. (1945) in cultured mice fibroblast, and it is an organelle found in all eukaryotic mammalian cells except matured red blood cells. ER is one of the vital cell organelles and a primary site for the biosynthesis of nearly 33% to 35% of all cellular proteins (Glembotski, 2008; Minamino and Kitakaze, 2010). ER is a cellular compartment responsible for several homeostatic responses including synthesis and post-translational modifications of secretory, luminal, and transmembrane proteins and folding and maturation of newly synthesized secretory and membrane proteins (Malhotra and Kaufman, 2011). In addition, ER serves as a site for steroid hormone synthesis, glucose concentration regulation, calcium homeostasis, and lipid metabolism (Liu et al., 2015).

The ER has two membrane subdomains, namely, rough and smooth ER. While the laminar structured rough ER is mainly responsible for synthesis, modification, folding, and processing of nascent polypeptide chains of secreted, transmembrane, and luminal proteins and Ca^2+^ signaling, the tubular structured, smooth ER is a central site for lipid biosynthesis and modification as well as Ca^2+^ signaling (Luchetti et al., 2017).

In eukaryotic cells, newly synthesized proteins need to undergo post-translational modification and proper folding in the ER lumen, as only correctly folded proteins are routed to the nucleus, Golgi apparatus, and cytosol. Protein folding is the process by which a linear polymer of amino acids acquires a unique 3D structure (the native conformation) capable of performing its cellular function. During the folding process, proteins often interact with molecular chaperones that bind to the hydrophobic regions of unfolded proteins to facilitate the folding process (Kitakaze and Tsukamoto, 2010). The post-translational modification processes include folding of the proteins into naïve 3D conformation, glycosylation, and disulfide bond formation (Chen et al., 2015). These complex cellular processes are governed by a highly regulated quality control system within the ER known as the UPR (Gardner et al., 2013), which is initiated in response to homeostatic de-arrangements, which trigger endoplasmic reticulum stress (ERS).

ERS is cellular stress within the ER, which was first described by Kozutsumi in 1998, when increased production of the 70-kDa glucose-regulated protein (GRP78) was induced in the presence of misfolded foreign proteins in the cytoplasm (Kozutsumi et al., 1988). Cells are susceptible to a range of environmental stimuli including radiation, hypoxia shock, heat, and oxidation, nutrient deprivation, energy disturbance, calcium depletion, oxidative stress (Kitakaze and Tsukamoto, 2010; Toko et al., 2010), and epigenetic alterations such as altered glycosylation, deoxyribonucleic acid (DNA) damage, DNA methylation, and histone modification (Schiano et al., 2015), which can result in aberrant protein folding and triggers ERS.

### ERS and the UPR Signaling

The UPR is a highly conserved adaptive system found in most eukaryotes and yeast. The UPR is a component of the ER adaptive system that comprises three principal signaling pathways, namely, inositol-requiring enzyme 1 (IRE1), PKR-like ER kinase (PERK), and activating transcription factor 6 (ATF6). These UPR sensors have two terminals, N-terminus located inside the lumen and C-terminus located in the cytosol, forming a bridge connecting the ER lumen to the cytosol (Minamino and Kitakaze, 2010). In addition, the UPR adaptive system is mediated by molecular chaperones and luminal enzymes, including the binding immunoglobulin protein (BiP) such as GRP78 and GRP94, calreticulin, and lectin calnexin, including protein disulfide isomerase (PDI) and Erp57, thiol-disulfide oxidoreductase (Malhotra and Kaufman, 2011). BiP/GRP78 is an Hsp70 family ER-resident molecular chaperone comprising an N-terminal ATPase and a C-terminal protein-binding domain and plays a mediating role in correct secretory and membrane protein folding, maintaining ER membrane permeability, and plays a critical role as a master regulator of ERS signaling (Wolfson et al., 2008).

In mammalian cells, protein synthesis is highly regulated to the changes induced by various environmental or pathological stimuli (Shi et al., 1998). Accumulation of misfolded proteins in the cytosol triggers a heat-shock response, which stimulates the transcription of genes encoding cytosolic chaperones to refold destabilized proteins. At the same time, accumulation of misfolded proteins in the ER initiates a signaling cascade extending from the ER to the nucleus that induces a comprehensive gene expression program to increase the protein folding capacity of the cell according to the physiologic demands (Kitakaze and Tsukamoto, 2010). Likewise, disturbance in a physiologic function of the ER leads to ERS and triggers activation of the evolutionarily conserved UPR to compensate for the damage. Furthermore, downstream of the three UPR signaling pathways, transcription factors are also activated, and UPR associated genes are upregulated. Eventually, activated UPR signaling mitigates ERS by suppressing synthesis of global proteins, inducing ER-associated degradation (ERAD) of unfolded/misfolded protein, and enhancing production of molecular chaperones, which facilitate proper protein folding (Gardner and Walter, 2011). However, persistent perturbation of the ER leads to malfunction within the network and chronic failure of the UPR adaptive system.

**Figure 1:**
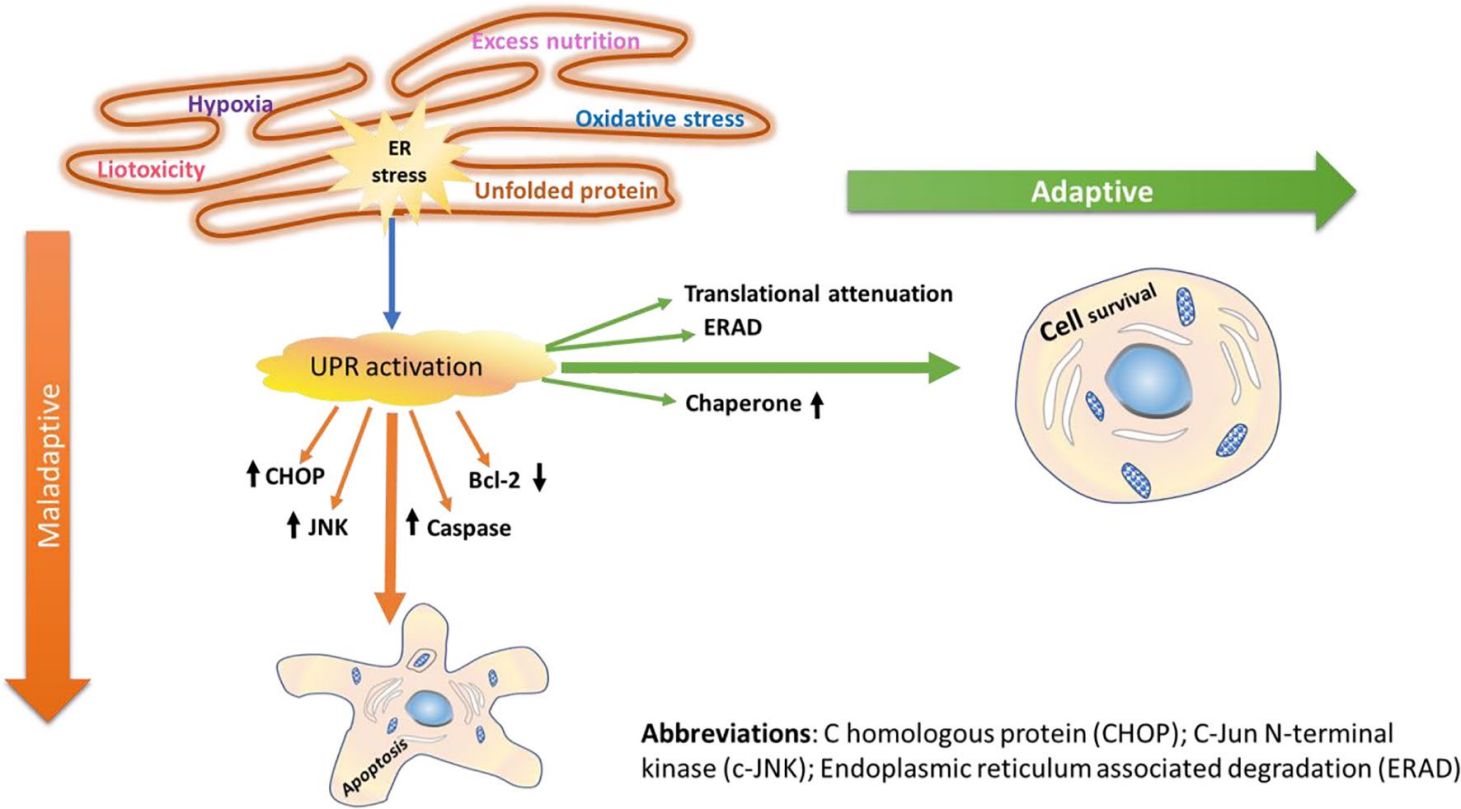
Adaptive and maladaptive mechanisms of UPR signaling. Following the onset of ERS in the cardiac system, cardiac-specific UPR is activated, and the three branches of signaling transducers (PERK, IRE1, and ATF6) are activated. Furthermore, downstream of the three UPR signaling pathways, transcription factors are also activated, and unfolded protein response (UPR) associated genes are upregulated. In general, activation of the UPR signaling networks initiates attenuation of the global protein translation, synthesis of folding chaperones and enzymes, and ERAD of misfolded/unfolded proteins in the ER lumen. Thus, through coordinated attenuation of global protein synthesis, upregulation of ER-resident chaperones, and activation of ERAD, the UPR often manages to resolve/restore acute homeostasis imbalances caused by various stimuli; thus, the cells survive (adaptive mechanism). The primary goal of the UPR activation is to avoid damages caused by the ERS through its adaptive functional role, which reestablishes the ER homeostasis, enhancing cell survival. However, intense ERS makes the cells succumb to apoptosis death (maladaptive mechanism). Once the ERS turns out to be persistent, transcription factors including CHOP, JNK, and caspases are activated, and members of the Bcl family such as Bcl-2 are suppressed, thereby eliciting programmed cell death.

When the ERS in the lumen is within the handling capacity of the UPR adaptive mechanism, the three branches of the UPR adaptive system (IRE1, PERK, and ATF6) are activated, leading to attenuation of global protein translation, synthesis of folding enzymes, and ERAD; and as a result, stress is resolved (Gardner and Walter, 2011). While synthesis of folding enzymes facilitates proper protein folding activity, attenuation of the global protein translation reduces the folding burden. Similarly, the ERAD eliminates aggregates of misfolded proteins, and this reduces the unfolded protein load within the network. As a result, the alterations caused by the ER are corrected and replenished without any further prolonged effects. However, chronic accumulation of unfolded/misfolded proteins followed by persistent disruption of the ER lumen may lead to malfunction of the UPR signaling to adapt itself to the changes caused by unfolded protein aggregates (Toko et al., 2010; Gardner et al., 2013). Consequently, this leads to latent apoptotic or necrotic cell death (Xu et al., 2005; Boyce and Yuan, 2006). Many lines of evidence have reported the role of ERS and initiation of UPR, which in chronic stress may lead to myocardial apoptosis, in the pathophysiology of various CVDs including heart failure, atherosclerosis, cardiac hypertrophy, and ischemic heart disease (Minamino and Kitakaze, 2010). Interestingly, the secreted and transmembrane proteins in the ER play important roles in maintaining myocardial health and disease (Doroudgar and Glembotski, 2012). In addition, some cardiac pathologies are implicated to impair secreted and membrane protein folding and disturb the homeostatic balance. For example, ERS-initiated apoptotic cell death was reported to play a favorable role in the pathophysiology of several chronic diseases including CVD and T2DM (Tabas and Ron, 2011). The adaptive and maladaptive mechanism mediated by the UPR signaling pathway is illustrated in **Figure 1**.

#### The Three Branches of UPR Signaling Effectors

The three main proximal effectors of UPR (PERK, IRE1, and ATF6) are activated with different strengths and at different time courses (Glembotski, 2014) and may act deferentially or synergistically. These effectors can serve both adaptive and maladaptive roles in many of the cell types. Under normal circumstances, all of these sensors exist in an inactive state through their maintained interaction with GRP78 *via* their luminal N-terminus (Oikawa et al., 2009). There is a consensus among the scientific community that the three UPR signaling pathway receptors are initiated in response to ERS, which can be triggered by various degrees of stimuli. The three arms of UPR transduction signaling networks detect the stress in the ER with their luminal domain and use their cytoplasmic domain to interact and communicate with transcriptional or translational machinery (Ron and Walter, 2007). Nevertheless, the exact mechanism how the three arms of ERS sensors detect and recognize the ER environment remains a subject of scientific debate. In the past, three mechanisms have been postulated to explain these phenomena, namely, the direct, semi-direct, and indirect (Kimata and Kohno, 2011; Parmar and Schröder, 2012). According to the indirect or negative regulation of ERS sensors, in unstressed cells, all the three ERS receptors are maintained inactive through their association with the ER chaperone BiP/GRP78. However, accumulated unfolded proteins in the ER lead to dissociation of GRP78 from the three ERS receptors, leading to subsequent activation of the three ERS sensors (Liu et al., 2000; Oikawa et al., 2009; Senkal et al., 2010). Contrary to this concept, it was reported that in yeast, the luminal domain of IRE1 detects accumulation of unfolded proteins through dissociation of BiP/GRP78 and direct interaction of IRE-1 receptor with unfolded proteins in the ER lumen (Kimata and Kohno, 2011; Parmar and Schröder, 2012). However, studies have revealed that unlike yeast IRE-1, the regulation of IRE-1 in mammals is dependent on the dissociation of BiP (Liu et al., 2000; Oikawa et al., 2009), implying that self-activation of IRE-1 may not be ligand dependent. In this regard, for the purpose and scope of this review, we are entirely confined to the indirect or negative regulation mechanism of the ERS sensors by the BiP/GRP78 chaperone proteins without any conflict with the rest of the proposed mechanisms.

##### PERK Signaling Pathway

PERK is one of the three main UPR signaling arms that are responsible for transducing ERS. First identified as a serine/threonine kinase in pancreatic islets of rat (Shi et al., 1998), PERK is a type I ER-resident transmembrane protein that mediates signal transduction during ERS (Harding et al., 1999). PERK is primarily involved in eukaryotic translation initiation factor 2 (eIF2α)-mediated translational attenuation and suppression of protein synthesis in the ER compartment (Sano and Reed, 2013). Under ERS, PERK dissociates from BiP/GRP78 and undergoes oligomerization and autophosphorylation, which leads to phosphorylation of the eukaryotic initiation factor eIF2α, a major substrate for PERK kinase activity (Kondratyev et al., 2007; Wolfson et al., 2008). Ultimately, this downstream signaling results in attenuation of global protein synthesis and translational activation of transcription factors (Shi et al., 1998) including ATF4 and C homologous protein (CHOP), which promote transcription of cell survival genes (Lei et al., 2017; Lumley et al., 2017). The translational arrest reduces the protein synthesis load, thereby maintaining the ER to its cellular homeostasis and facilitating efficient ER protein folding and assembly.

**Figure 2:**
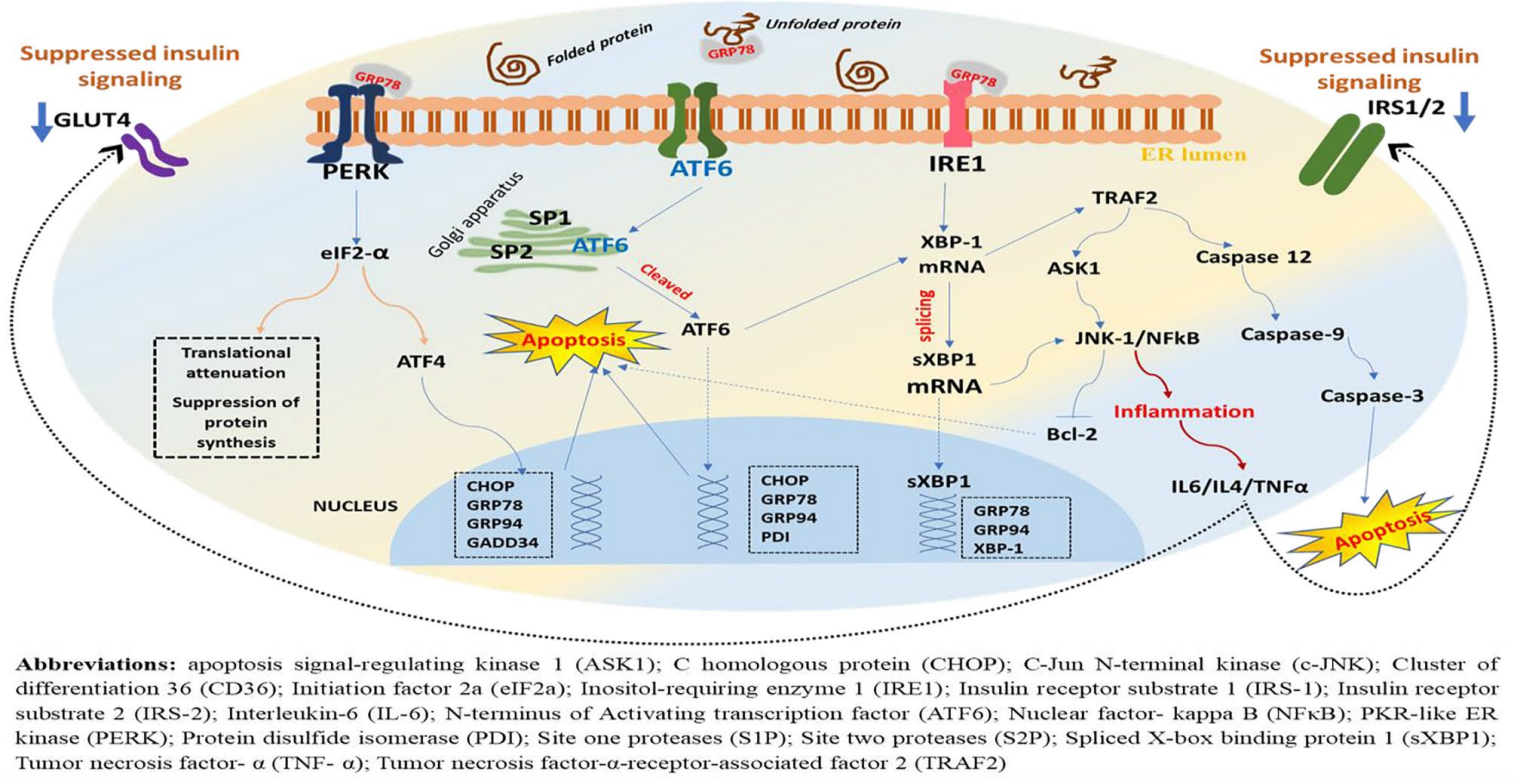
A schematic representation of downstream effectors, target genes, and possible outcome in the ERS-activated UPR. Under ERS, dissociation of GRP78 from its luminal domain leads to oligomerization and autophosphorylation of PERK, ensuing its kinase and endoribonuclease activities. As a result, the α subunit of eukaryotic initiation factor 2 (eIF2) undergoes phosphorylation, resulting in translational attenuation characterized by a reduction in protein biosynthesis. Parallelly, this downstream phosphorylation of eIF2 leads to increased expression of ATF4 and translocation into the nucleus where it binds to the UPR element (UPRE) resulting in transcriptional modification of UPR target genes including the proapoptotic transcription factor, C homologous protein (CHOP), GRP78, GRP94, and GADD34, while attenuating global translational process, but PERK phosphorylation also inhibits transcription of I kappa B alpha (IκBα), leading to hyperactivation of NF-κB and increased production of inflammatory cytokines (**Figure 4**). On the other hand, ERS leads to autophosphorylation of IRE-1, leading to excision and splicing of its substrate, XBP1 mRNA. Consequently, this results in spliced XBP1 protein (sXBP1), which translocates into the nucleus, and this will upregulate genes for protein folding enzymes secretion and ER-associated protein degradation (ERAD). Noteworthy, the ATF6 is activated following PERK and prior to IRE1. The GRP78 dissociates from ATF6 and recruited to luminal protein aggregates resulting in translocation of ATF6’s cytosolic fraction to the Golgi, where it is spliced and proteolyzed by site one proteases (S1Ps) and site two proteases (S2Ps). Eventually, this leads to the release of the cytosolic domain of ATF6 and entry into the nucleus where it sequesters with the ERS response element (ERSE) resulting in the activation of UPR target genes including XBP1, CHOP, and GRP78/BiP, GRP94, PDI. However, chronic ERS, which may result from persistent perturbation of the ER, can lead to malfunction within the network and failure of the UPR adaptive system. And as a result, the PERK-mediated post-translational attenuation is inhibited and further accumulations of unfolded protein aggregates, leading to initiation of C homologous protein (CHOP)-mediated apoptotic deaths of the cells.

The role of PERK not is limited to the activation of eIF2α/ATF4-mediated attenuation of global protein synthesis, translational activation of transcription factors, and apoptotic event *via* CHOP but also encompasses ROS detection and ER and mitochondrial tethering (Thoudam et al., 2016). Following eIF2α phosphorylation, the mRNA that encodes ATF4 is translated more efficiently and leads to increased expression of ATF4 (Glembotski, 2008). As a result, ATF4 is translocated into the nucleus where it binds to the unfolded protein response element (UPRE), resulting in transcriptional modification of UPR target genes including the proapoptotic transcription factor, CHOP (Lei et al., 2017). It is noteworthy that the ATF4 transcriptional activity in the early stage of ERS induces prosurvival pathways, whereas in the late phase where ERS is prolonged, it initiates the proapoptotic transcriptional programs through increased expression of the proapoptotic protein CHOP (Groenendyk et al., 2010). On the other hand, phosphorylation of PERK pathways (eIF2α/ATF4/CHOP) attenuates global translational process and inhibits transcription of I kappa B alpha (IκBα), leading to hyperactivation of nuclear factor-kappa B (NF-κB) (Liu et al., 2015). However, if the ERS is restored, activation of GADD34 (growth arrest and DNA-damage-inducible protein 34) follows, and this dephosphorylates eIF2α *via* negative feedback (Basseri and Austin, 2012), resulting in attenuation of translational repression exerted due to ERS (**Figure 2**).

##### The IRE-1 Signaling Pathway

IRE-1 is also a transmembrane protein conserved in all eukaryotic cells and plays significant roles in the UPR adaptive system. The IRE-1 is a transmembrane protein that encodes a type 1 ER-resident transmembrane protein and is present as an inactive monomer until it undergoes autophosphorylation and becomes an active oligomer forming clusters on the ER (Li et al., 2010). IRE1 has protein kinase and ribonuclease domains that extend through its cytoplasmic region. Under normal conditions, the IRE-1 remains bound to GRP78 in the ER membrane, forming an inactive complex. However, in response to ERS and dissociation of GRP78 from the ER lumen, IRE-1 undergoes homodimerization and autophosphorylation and is released as an active RNase of IRE-1 with conformational change (Liu et al., 2000; Oikawa et al., 2009). Upon autophosphorylation, unlike the PERK receptor, IRE-1 exhibits a novel endoribonuclease activity (Harding et al., 1999; Glembotski, 2008), which leads to excision and splicing of its substrate, the 26-nucleotide sequence XBP1 mRNA (Yoshida et al., 2001). The splicing of XBP1 mRNA, which takes place in the cytosol, generates a transcript with a new open reading frame that encodes the expression of an active form of XBP1 (Yoshida et al., 2001). This results in spliced XBP1 protein (sXBP1) with 5′ and 3′ mRNA fragments that enhance internal ribosome entry site (IRES)-dependent translation of XBP1 mRNA and leads to transactivation of UPR target proteins including CHOP, XBP1, endoplasmic reticulum oxidoreductin 1 (ERO1), P58IPK, ER degradation-enhancing α-mannosidase-like protein (EDEM), and RAMP-4 (Toth et al., 2007).

The XBP1 mRNA expression is dependent on the activation of the ATF6 receptor protein. Translocation of the spliced XBP1 mRNA into the nucleus facilitates the upregulation of genes that are crucial for protein folding and secretion, and ERAD (Basseri and Austin, 2012; Thorp, 2012). The translocated sXBP1 mRNA binds to the promoter region of the ERS response element (ERSE) I and II (ERSE-I: CCAAT(N9) CCACG; ERSE-II: ATTGG(N1) CCACG) and the mammalian UPR element (mUPRE: TGACGTGG/A) to regulate a variety of UPR-related genes that are essential to induce degradation of malfunctioned cells (Minamino and Kitakaze, 2010; Luchetti et al., 2017). On the other hand, IRE-1 collaborates with TRAF2 to activate protein kinases that are associated with inflammatory response and cellular apoptosis, particularly the apoptosis signal-regulating kinase 1 (ASK1), which leads to activation of JNK (Kim et al., 2008) (**Figure 2**).

##### ATF6 Signaling Pathway

ATF6 is an ERS-regulated transmembrane transcription factor with an N-terminal domain in the cytosol and a C-terminal in the ER lumen (Zhang and Kaufman, 2004), which activates the transcription of ER molecular chaperones to sense ERS. In addition to the two isoforms, ATF6α and ATF6β, there are other ATF6-related membrane proteins, namely, Oasis, Luman, CREB4, CREB-H, and BBF2H7, all of which are ER transmembrane proteins that are cleaved and translocated to the nucleus under ERS (Glembotski, 2008). Although ATF6 does not undergo oligomerization or autophosphorylation (Zhang and Wang, 2012), the activation mechanism is partially similar to that of PERK and IRE-1, which is through dissociation of BiP/GRP78. In an experiment, (Shen) described the dissociation of BiP/GRP78 during ERS, which allows ATF6 to be relocated to the Golgi complex, as follows. Under ERS conditions, GRP78 dissociates from the N-terminus of ATF6, and this initiates the translocation of ATF6 from the ER lumen to the Golgi complex (Shen et al., 2002), suggesting that BiP/GRP78 dissociation from ATF6 in response to ER stress is detrimental for the downstream activation of ATF6-mediated signaling pathway. Translocated to the Golgi, ATF6 is spliced and proteolyzed by the Golgi resident site 1 protease (S1P) and site 2 protease (Ye et al., 2000). The fact that the proteases (SP1 and SP2), which catalyze the cleavage of ATF6, are located in the Golgi suggests that ATF6 translocation to Golgi is critical for its activation (Morishima, 2011). Consequently, the cleavage of ATF6 at a juxta-membrane site leads to its release into the cytosol (Xu et al., 2005). The cytosolic 50-kDa soluble fragment of ATF6 is then translocated into the nucleus where it sequesters to ATF/cAMP response elements (CRE) and endoplasmic reticulum stress response element (ERSE-I) (Sano and Reed, 2013). In turn, this leads to transcriptional regulation and activation of UPR target genes including XBP1, CHOP, and GRP78/BiP (Basseri and Austin, 2012; Thorp, 2012).

ATF6 is a major inducer of the expression of genes encoding molecular chaperone and elements of ERAD (Adachi et al., 2008). In the heart, there are two ATF6-inducible genes, namely, RCAN1 and Derl3, which are shown to be adaptive and encode proteins that attenuate increased protein-folding demand and facilitate degradation of unfolded proteins (Glembotski, 2014). According to a study by Toko, Takahashi, et al. data from *in vivo* experimental study conducted using mice indicated that ATF6 activity was increased in a mouse model of myocardial infarction. Furthermore, treatment of myocardial infarction-induced mouse with ATF6 inhibitor, 4-(2-aminoethyl)benzenesulfonyl fluoride, further reduced cardiac dysfunction and increased mortality rate. However, suppression of ATF6 with pharmacological agent improved the cardiac function, suggesting the critical role of ATF6 in both pathological and physiological states (Toko et al., 2010).

In response to ERS, all the three UPR receptors, PERK, IRE1, and ATF6, are activated to mediate adaptive and/or maladaptive functions. Although ATF6 is believed to predominantly play an adaptive role in many of the cell types, studies showing the role of ATF6 in the maladaptive UPR signaling pathway *via* activation of the apoptotic mechanism are emerging. For example, a study conducted on myoblast revealed the activation of ATF6 in apoptotic myoblasts during differentiation, suggesting the maladaptive function of ATF6 (Morishima, 2011). Although much details remain, the finding of this and other relevant studies has highlighted that ATF6 may trigger apoptosis during persistent ER stress *via* activation of downstream distal effectors including CHOP, JNK, and the proapoptotic Bcl-2 family proteins (Senkal et al., 2010) (**Figure 2**).

Importantly, the ER and ERS-initiated UPR signaling pathway have emerged as critical aspects of diverse physiological and pathological processes in many cell types. Perturbations caused by protracted stress, abnormal protein synthesis, and hypercholesterolemia may disrupt the protein folding capacity of ER lumen, leading to accumulation of unfolded/misfolded proteins, which eventually causes deleterious effects, designated as ERS, on the physiologic function of the cells (Zhang and Kaufman, 2004). As a first line of defense, ERS-initiated activation of UPR signaling transduction elicits protection against moderate ERS through coordinated function of the three proximal arms of UPR and ultimately regulate the translation and transcription of various proteins and enhance the expression of genes encoding molecular chaperones and folding enzymes by enhancing ER folding capacity and/or by attenuating protein translation. However, in conditions of persistent stress, the UPR signaling initiates programmed cell death/apoptosis mechanism to eliminate unhealthy cells and to suppress the spread of their toxic effects (**Figure 2**).

### ERS and Inflammation in CVD

#### Inflammation

The ability of eukaryotic organisms to survive is dependent on their ability to fight and defend themselves from infectious organisms, heal damage or injury caused, and reserve energy for times of high demand. Inflammatory responses triggered by the collaborated activity of the innate and adaptive immunity are among such natural defensive systems through which multicellular organisms overcome infectious agents or deviant environmental stimuli. Metabolic and immune systems are among the homeostatic components that have evolved to be closely linked and codependent (Wellen and Hotamisligil, 2005). Thus, the integration of metabolism and immunity determines the maintenance of a complete immune system and good health. However, under aberrant conditions, for example, in nutritional excess or poor nutrition, this integration can trigger deleterious health effects.

Inflammation is an adaptive mechanism initiated in response to cell damage caused by endogenous or exogenous stimuli such as infectious microorganisms, trauma, or chemical agents (Kawasaki et al., 2012). In the normal physiological state, acute inflammation acts as an adaptive system to get rid of a stimulus. However, chronic inflammation resulting from repeated injury and repair leads to damage and malfunctions to the tissue. Inflammation is considered a crucial element in the development and progression of chronic metabolic diseases including diabetes and CVD (Thoudam et al., 2016). Chronic inflammation of the vascular system is implicated in the pathophysiology of cardiac dysfunction. CVD is a chronic inflammation of the lining of the blood vessel, which results due to transendothelial infiltration of cholesterol-rich and atherogenic Apo-B lipoproteins from the plasma into the intima. Consequently, these infiltrated lipoproteins are retained in the sub-endothelia, facilitating macrophage and T-cell migration and sequestering with one another and cells of the arterial wall, an event that promotes atherosclerosis (Nakayama and Wang, 2010).

Unlike the classical inflammation with typical cardinal features such as pain, heat, swelling, and redness, chronic low-grade inflammation, also known as metabolic inflammation, is a form of inflammation often triggered by adiposity/obesity (van Diepen et al., 2013). This type of inflammation is associated with the development of metabolic syndrome including atherosclerosis, hypertension, and prothrombin (Emanuela et al., 2012), which are risk factors for the development of T2DM and CVD. Although the mechanism of chronic low-grade inflammation is not precisely known, the increased translocation of lipopolysaccharide (LPS) or endotoxin from gut to circulation, which causes metabolic endotoxemia, is speculated. In addition, animal studies have shown that diets high in saturated fatty acids and cholesterol activate inflammatory processes in adipose tissue, liver, and vasculature (van Diepen et al., 2013).

Several studies have documented a strong association between obesity and chronic inflammation. Chronic low-grade systemic and local inflammation that is triggered in condition of nutritional excess is implicated in various metabolic disorders including T2DM and CVD. Obesity itself is characterized by an inflammatory response in which crucial mediators of inflammation like tumor necrosis factor-alpha (TNF-α) show significant patterns of expression in both animal models and humans. In addition, data obtained from transcriptional profiling studies showed that inflammatory and stress-response genes are highly upregulated in adipose tissue of obese animals (Wellen and Hotamisligil, 2005).

Continuous deposition of lipids and expansion or hypertrophy of adipose tissue (AT) stimulates adipose tissue macrophages (ATM) and other infiltrating macrophages to generate excess inflammatory cytokines including interleukin-6 (IL-6), TNF-α, c-Jun N-terminal kinase (c-JNK), and NF-κB, resulting in metabolic inflammation (van Diepen etal., 2013; Chen et al., 2015). In addition, the presence of excess ROS can also trigger oxidation of low-density lipoprotein (LDL), which is taken up by macrophages *via* scavenger receptors, leading to the development of atherogenesis (Sozen and Ozer, 2017), a common feature of CVD. These events are considered contributing factors for obesity-associated inflammation in which pro-inflammatory cytokines like TNF-α and IL-6 are increasingly expressed and anti-inflammatory markers such as adiponectin are downregulated.

Pattern-recognition receptors (PRRs) including Toll-like receptors (TLRs) and the receptor for advanced glycation end-products (RAGE) are implicated in the pathophysiology of obesity-related inflammation and associated complications. The PRRs are components of innate immunity and are activated in response to aberrant conditions such as over-nutrition. TLRs comprise different subfamilies that have unique features of sensing pathogens and detecting tissue injury *via* the specific pathogen-associated molecular patterns (PAMPs) and the danger-associated molecular patterns (DAMPs) (Ota, 2014).

#### ERS-Mediated Inflammation

**Figure 3:**
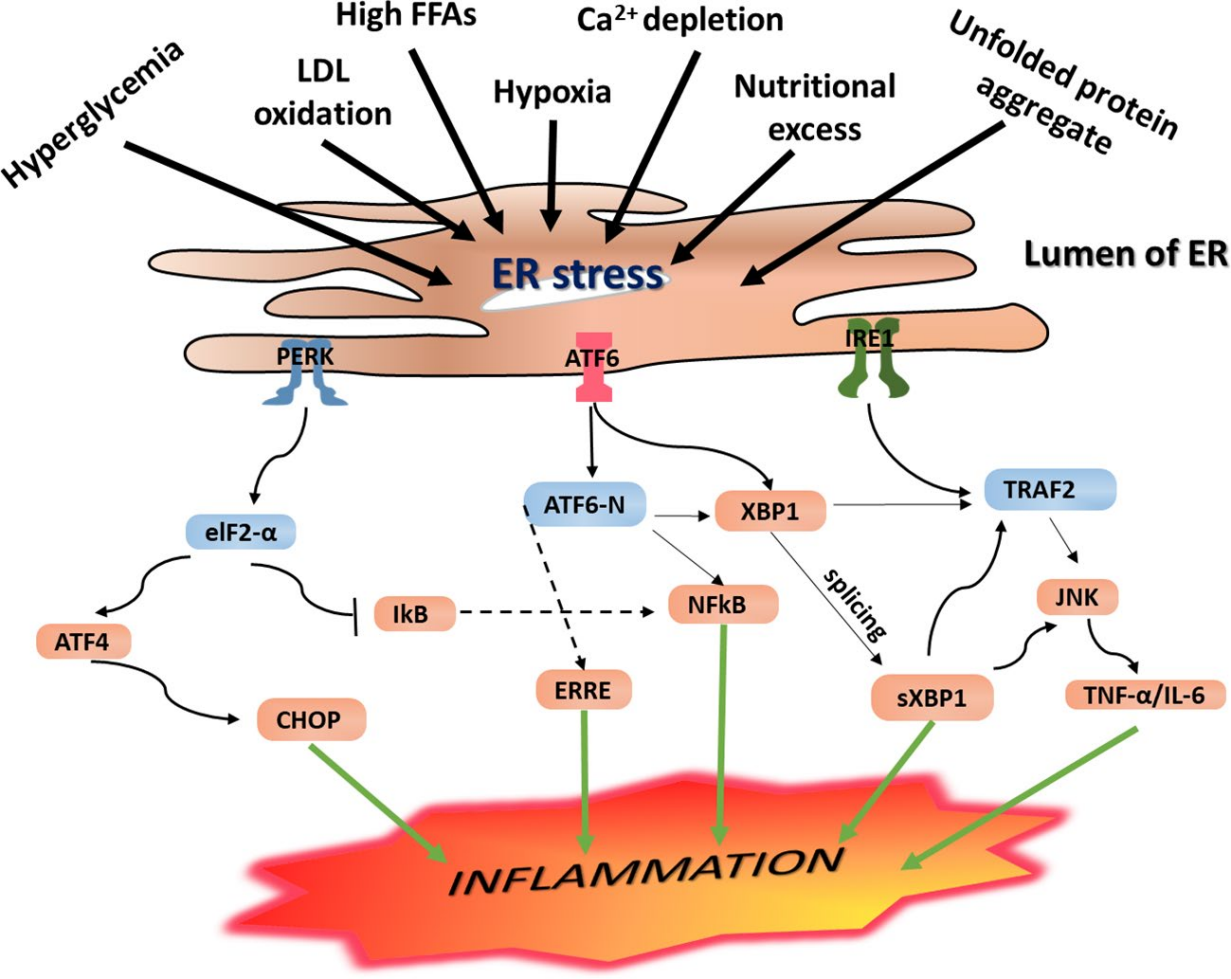
Endoplasmic reticulum (ER) stress-mediated inflammation. Under ERS condition, the UPR is activated, and this leads to activation of the three principal UPR signaling transmembrane receptor proteins including IRE-1, PERK, and ATF6. Activation of the IRE-1 leads to the splicing of the mRNA of a transcription factor XBP1 and subsequent expression of sXPB1, a highly active transcription factor for the release of ER-resident enzymes and molecular chaperones. As a result, it leads to activation of NF-κB and CHOP resulting in increased expression of proinflammatory gene products. Likewise, activated IRE-1 recruits TRAF2, and this complex causes activation of downstream signaling of kinases including JNK and NF-κB, which induce production of inflammatory cytokines and trigger inflammation. These inflammatory kinases then phosphorylate and activate downstream mediators of inflammation. Phosphorylation of PERK/eIF2a downstream signaling pathway results in uncoupling of NF-κB from IkB. As a result, NF-κB translocates into the nucleus where it activates expression of genes encoding proinflammatory cytokines including IL-1, IL-6, and TNF-a, resulting in persistent inflammatory response. On the other hand, autophosphorylation of PERK initiates activation of eukaryotic initiation factor 2 (eIF2), which further undergoes phosphorylation resulting in translational attenuation of protein synthesis. Similarly, this downstream phosphorylation of eIF2 leads to increased expression of ATF4 and translocation into the nucleus where it binds to the UPRE resulting in transcriptional modification of CHOP, a proapoptotic gene transcription factor that initiates inflammation as well as apoptosis. IRE-1 recruits TRAF2 and causes activation of downstream signaling of kinases including JNK and NF-κB, inducing the production of inflammatory cytokines. PERK phosphorylates eIF2α, which leads to the activation of NF-κB and CHOP to further promote the expression of the inflammatory gene. ERS leads to dissociation of TRAF from TRAF2-procaspase 12 complex, which is located on the ER membrane, leading to activation of caspase 12. At the same time, IRE1–JNK complex recruits TRAF2 leading to the formation of the IRE1–JNK–TRAF2 complex. The ATF6 pathway also activates NF-κB, further intensifying the expression of inflammatory genes, which secrete more cytokines.

As discussed in Section 3.2, when ERS is induced, the UPR signaling is activated, resulting in increased expression of GRP78 and phosphorylation of IRE-1, PERK, and ATF6 signaling receptors. Through activation of these UPR signaling networks, ERS is resolved. However, if the UPR adaptive mechanisms fail to restore the imbalance caused by ERS to a physiologic homeostasis, the IRE-1α-dependent apoptotic signaling pathway, which occurs *via* diverse pathways, is inevitable. As illustrated in **Figure 3**, phosphorylation of IRE-1α leads to TRAF2 recruitment causing activation of downstream signaling of JNK and NF-κB, leading to the production of proinflammatory cytokines (Chen et al., 2015). Additionally, phosphorylated IRE-1α interacts with a mitogen-activated protein kinase (MAPK), ASK1, which eventually phosphorylates JNK (Minamino and Kitakaze, 2010).

As discussed in the section *PERK Signaling Pathway*, PERK phosphorylates eIF2α, and downstream signaling of this pathway upregulates inflammatory genes and increases secretion of proinflammatory proteins including TNF-α and IL-6, resulting in persistent inflammation. In parallel, autophosphorylation of PERK initiates activation of eukaryotic initiation factor 2 (eIF2), which further undergoes phosphorylation and results in translational attenuation of protein synthesis. This downstream phosphorylation of eIF2 leads to increased expression of ATF4 and translocation into the nucleus, where it binds to the UPRE resulting in transcriptional modification of CHOP, a proapoptotic gene transcription factor that initiates inflammation as well as apoptosis. IRE-1 recruits TRAF2 and causes activation of downstream signaling of kinases including JNK and NF-κB, inducing the production of inflammatory cytokines. PERK phosphorylates eIF2α, leading to activation of NF-κB and CHOP to further promote the expression of inflammatory genes (**Figure 3**).

ERS and activation of the UPR signaling play a crucial role in the development of CVDs. For example, UPR activation is implicated in ischemic myocardium, cardiac hypertrophy, and heart failure. Likewise, increased expression of CHOP and caspase 12 is implicated in the progression of cardiac hypertrophy to heart failure and cardiomyocyte death (Kitakaze and Tsukamoto, 2010). Many lines of evidence indicated the important role of ERS in the development and progression of CVDs, but there remains much to understand the molecular mechanisms of ERS and chronic activation of UPR signaling pathway in the pathogenesis of CVDs. Although most of the knowledgeable discussed above are well established in systems other than CVS, much remains to learn the CVS-targeted underlying molecular mechanisms of ERS-induced CVDs *via* activation of the UPR signaling pathways.

### ERS and Insulin Resistance

#### Insulin Resistance

Insulin, secreted by the pancreatic beta cells, is a key regulatory enzyme in carbohydrate–lipid metabolism. Insulin plays a significant role in maintaining energy balance in a physiological state and helps decrease circulating blood glucose by increasing cell permeability to monosaccharides, amino acids, and fatty acids. In addition, insulin facilitates metabolic processes such as glycolysis, the pentose phosphate cycle, and glycogen synthesis in the liver, which eventually leads to reduced circulating blood glucose. Metabolic functions of insulin include facilitating glucose infusion into metabolic tissues, stimulating the synthesis of fatty acids and glycogen, promoting mitochondrial function, promoting microcirculation, and cell proliferation (Ye, 2013). Insulin also helps downregulate essential gluconeogenic enzymes in the liver, promotes triglyceride deposition by accelerating glucose degradation *via* glycolysis and synthesis of free fatty acids, and inhibits lipolysis in fat cells (Stafeev et al., 2017).

**Figure 4:**
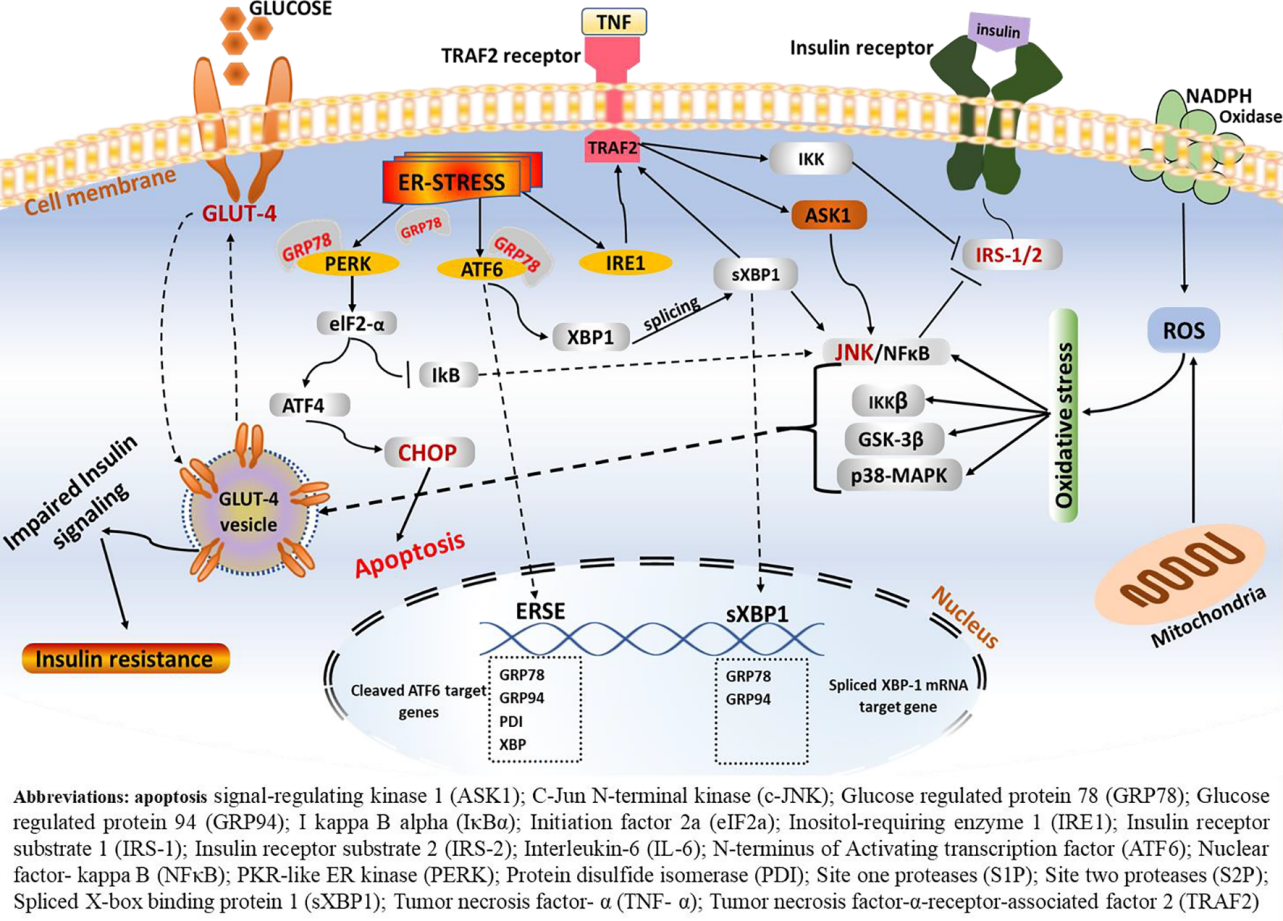
ERS-mediated insulin resistance. Following the onset of ERS, the GRP78 BiP dissociates from the three central signaling receptors of the UPR (PERK, ATF6, and IRE-1). On the one hand, IRE1–JNK complex recruits TRAF2 leading to the formation of the IRE1–JNK–TRAF2 complex. The IRE1–JNK–TRAF2 complex has an effect on ASK1 activation, which may induce JNK phosphorylation, ultimately leading to ablation in insulin receptors and results in insulin resistance. Activated IRE-1 recruits TRAF2, and this complex causes activation of downstream signaling of kinases including JNK and NF-κB, which induces production of inflammatory cytokines, which trigger inflammation. These inflammatory kinases then phosphorylate and activate downstream mediators of inflammation. Consequently, IRE-1-mediated JNK activation disrupts insulin receptor signaling and results in insulin resistance. Phosphorylation of one of these arms, namely, the IRE1α, enhances recruitment of TRAF2, which further activates the c-JNK. In turn, c-JNK activation triggers increased production of proinflammatory markers, which are believed to alter insulin receptor and leads to insulin resistance. On the other hand, phosphorylation of IRE-1α fosters phosphorylation of IRS-1 through JNK-dependent serine and thus inhibits insulin receptor signaling.

Activation of the insulin receptor tyrosine kinase, which leads to phosphorylation of insulin receptor substrate (IRS) on multiple tyrosine residues, is the primary molecular signaling node for insulin action (Kahn and Flier, 2000). Insulin binds to insulin receptors on the surface of insulin-responsive cells and stimulates insulin-responsive receptors and other substrates including IRS-1 and IRS-2. Interaction of insulin to IRS leads to phosphorylation of IRS, thus initiating downstream insulin signaling cascades. Binding of insulin to its receptor, receptor phosphorylation, tyrosine kinase activity, and phosphorylation of IRS are reported in both muscle cells and adipocytes (Kahn and Flier, 2000). In addition, the insulin-dependent translocation of glucose transporter 4 (GLUT4) in the skeletal muscle and fat cells and increased expression of glucose transporter 1 (GLUT1) in vascular endothelium cells play a major role in maintaining the physiological function of insulin (Stafeev et al., 2017). When this physiologic state is perturbed, however, insulin production is impaired and leads to reduced insulin secretion and elevated blood glucose profile by raising circulating concentrations of glucose, free fatty acids, and other nutrients. In most cases, such failure is the outcome of direct impairment in the insulin signaling pathway, mainly due to a disturbance in the IRS and/or downregulation of the insulin-responsive GLUT4 (**Figure 4**). These functional defects can have critical metabolic consequences in glucose homeostasis and lead to reduced cellular responsiveness to insulin action, thereby resulting in a condition known as insulin resistance (Eckel et al., 2011).

Insulin resistance is characterized by hyperinsulinemia and hyperglycemia, decreased postprandial glucose infusion rate, high levels of glycosylated hemoglobin (HbA1c), glucose intolerance, hyperlipidemia, elevated plasma inflammatory markers, and decreased production of adiponectin (Ye, 2013). Insulin resistance is manifested by a decrease in insulin-stimulated glucose transport and by altered hepatic glucose output, mainly *via* the IRS/PI-3-kinase/PKB signaling arm. In response to elevated blood glucose level, pancreatic β-islets tend to produce more insulin to overcome the disturbance through positive feedback response, favoring a condition known as hyperinsulinemia. Hyperinsulinemia is a common feature of insulin resistance and T2DM. It is widely accepted that obesity-induced metabolic perturbations promote insulin resistance and β-cell dysfunction (Eckel et al., 2011). Excess abdominal adipose tissue has been shown to release increased Fas, which directly affect insulin signaling. In their review, Qatanani and Lazar (2007) have described that overweight people have impaired ability to uptake circulating glucose from the bloodstream into fat and muscle cells and to reduce the conversion of glycogen into glucose in the liver, leading to elevated blood glucose level.

Several studies have inferred associations between hyperglycemia diabetic cardiomyopathy *via* increased supply of free fatty acids (FFAs) to the cardiomyocytes. Johnson et al. (2017) hypothesized that chronically elevated blood glucose level triggers enhanced transportation of FFAs to cardiomyocytes to a concentration that overwhelms the rate of cellular β-oxidation. This imbalance may lead to accumulation of triglycerides, triggering lipotoxicity and left ventricular dysfunction (Johnson et al., 2017). Clearly, the pathophysiology of insulin resistance may include many cellular pathways such as the inflammatory, neural, and endocrine pathways (Qatanani and Lazar, 2007) and deregulation of caloric intake and expenditure in the central nervous system, systemic lipid metabolism, and perturbation in insulin and glucose signaling in the periphery (Hotamisligil, 2010). However, the precise molecular mechanisms are subject to further research.

#### ERS-Mediated Insulin Resistance

Disturbance of immune signaling and ER perturbation are integrating factors influencing metabolic regulation and emergence of metabolic disease. Infiltration of immune cells and alterations in inflammatory signaling pathways in adipose tissue are characteristic features in obesity-induced insulin resistance. Moreover, the ERS and associated perturbation of the immune signaling has long been documented as critical factors that cause metabolic dysregulation and emergence of metabolic disease including insulin resistance and CVD (Hotamisligil, 2010).

Studies have shown that obesity-induced ERS results in insulin resistance and T2DM, mainly through activation of the JNK terminal, a mediator that leads to an alteration in insulin signaling. Several studies have highlighted this phenomenon in the pathophysiology of obesity-mediated insulin resistance. According to Wellen and Hotamisligil (2005), cells exposed to one of the proinflammatory cytokines, such as TNF-α or high FFAs, have stimulated inhibition of the serine residues of IRS-1, thus barring downstream insulin signaling/insulin action. On the other hand, following stimuli such as ERS, elevated level of cytokines, and high fatty acids, JNK phosphorylates and associates with the IRS-1 and impairs the sensitivity of the receptor to insulin action (**Figures 2** and **4**).

Low-grade chronic inflammation, also known as metainflammation, is often triggered in response to an excess nutritional state such as high circulating glucose and saturated fatty acids and is an emerging mediator of obesity and insulin resistance. Commonly, this type of metabolic inflammation is triggered *via* PRR families such as TLRs, inflammasome, RAGE, and nucleotide oligomerization domain (NOD) activation. TLR2 and TLR4 are highly implicated in obesity-associated inflammation, and insulin resistance as activation of these receptors leads to elevated production of inflammatory cytokines and recruitment of T-cells and macrophages (Ota, 2014).

Studies have shown that excess nutrition/obesity activates the immune system, specifically innate immunity through PRRs and leads to recruitment of T-cells and macrophages, subsequently desensitizing the insulin signaling pathways and leading to the development of insulin resistance, a precursor of T2DM (Mcfarlane et al., 2001). Adipose tissue macrophages (ATMs) are primary sources of proinflammatory cytokines such as TNF-α and IL-6, which suppress insulin action and induce the development of insulin resistance.

In his seminal article, Hotamisligil has extensively discussed the role of JNK, particularly the JNK1 isoform, as a central mediator in the pathophysiology of insulin resistance mainly by IRS-1 serine phosphorylation and subsequent inhibition of insulin actions, through activation of inflammatory signaling cascades (Wellen and Hotamisligil, 2005; Hotamisligil and Erbay, 2008). IRS-1 and IRS-2 are essential substrates for the insulin receptor tyrosine kinases (Chen et al., 2015; Liang et al., 2015) in insulin signaling pathways. Thus, ablation of the IRS-1/2 receptors by overexpression of inflammatory molecules suppresses the insulin signaling pathway and results in insulin resistance.

Production of proinflammatory cytokines and suppression of IRS-1 has been documented as a primary progressive signaling node in the development of insulin resistance. JNK activation and downstream activation of inflammatory signaling pathways are common features in ERS. Although the complete mechanism is yet to be elucidated, under ERS, the GRP78/BiP dissociates from the three central signaling receptors of the UPR including PERK, ATF6, and IRE-1. Phosphorylation of IRE-1α enhances recruitment of TRAF2, which further activates the c-JNK (Chen et al., 2015; Liang et al., 2015). In turn, c-JNK activation triggers increased production of pro-inflammatory markers that are believed to alter insulin receptor levels and lead to insulin resistance. As illustrated in **Figure 2**, activated IRE-1 recruits TRAF2, and this complex causes activation of downstream signaling of kinases including JNK and NF-κB, which induces production of inflammatory cytokines. Consequently, IRE-1-mediated JNK activation disrupts insulin receptor signaling and results in insulin resistance. This is in line with data from experimental studies conducted on mice wherein significant protection against insulin resistance and T2DM following JNK1 gene knockout is reported (Hotamisligil and Erbay, 2008). Elevated level of fatty acids, high blood glucose, ERS, and mitochondrial dysfunction and increased production of inflammatory cytokines result in increased inhibitory actions of Ser/Thr phosphorylation of IRS-1/2 *via* activation of JNK, IkB kinase (IKK), mammalian target of rapamycin (mTOR) C1/S6K, and MAPK (Boucher et al., 2014). ERS leads to suppression of insulin receptor signaling, which leads to diminished insulin signaling through overactivation of JNK and subsequent serine phosphorylation of IRS-1. Mice deficient in XBP1 mRNA develop insulin resistance. Upregulation of sXBP-1 mRNA modulates ERS and suppresses JNK, which enhances insulin signaling, indicating the central role of ERS in insulin resistance and T2DM (Özcan et al., 2004).

In studies conducted on heterozygous IKKβ and whole-body JNK1-deficient mice, moderate protection against high-diet-induced insulin resistance was observed (Ota, 2014). This supports the notion that the IKKβ/NF-κB and JNK pathways are involved in the development of insulin resistance and are mechanisms that connect inflammation with insulin resistance. Besides, activation of the IKKβ/NF-κB pathway dephosphorylates IRS-1 through increased expression of PTP1B (Kim et al., 2008; Zabolotny et al., 2008). Akt also has a central role in regulating insulin action. Akt phosphorylates and activates IKK, which leads to activation of NF-κB (Boucher et al., 2014), resulting in diminished insulin signaling.

Akt is implicated as a central mediator of various insulin actions. Akt mediates insulin actions mainly by regulating apoptotic and survival proteins, cell cycle regulating proteins, and transcription factors. Among others, Akt phosphorylates murine double minute 2 (Mdm2), leading to inhibition of p53-mediated apoptosis. The Bax, Bad, and caspase-9 proapoptotic mediators are phosphorylated and inhibited by promoting cell survival. It also phosphorylates and activates IKK, which leads to activation of NF-κB. Endothelial nitric oxide synthase (eNOS) is also phosphorylated by Akt, thereby enhancing the production and release of nitric oxide (NO), a prominent vasodilator and anti-inflammatory molecule (Boucher et al., 2014). However, when this physiologic balance is perturbed, it may lead to diminished insulin signaling, resulting in insulin resistance. However, a better understanding of the etiological factors involved and the complete molecular mechanisms behind insulin resistance and its association with CVD would inevitably be helpful in the effort to combat cardiovascular pathologies and T2DM-associated comorbidities.

### Oxidative Stress and CVD

Oxidative stress is characterized by overproduction and accumulation of ROS beyond the antioxidant capacity of living cells to scavenge harmful ROS. In living cells, free radicals, predominantly in the form of ROS and reactive nitrogen species (RNS), are fundamental biochemical components and are typical by-products of cellular metabolic processes (Pavithra and Vadivukkarasi, 2015). Beyond their physiological roles, however, ROS are also the primary etiological factors and characteristic features of several chronic diseases including CVD. Interestingly, in the cardiovascular system, ROS acts as messenger in specific signaling pathways involved in regulating cardiovascular homeostasis (Csányi and Miller, 2014; Cervantes Gracia et al., 2017). However, the presence of excess ROS deters pathophysiologic components in several oxidative stress associated diseases such as cardiovascular diseases (CVD), rheumatoid arthritis, cancers, ulcerative colitis, and neurodegenerative disorders (Pavithra and Vadivukkarasi, 2015).

Several studies have elucidated the effects of ROS-induced oxidative stress in CVD and further demonstrated ROS as a significant contributing factor in the pathogenesis of CVD. Accumulating evidence indicates a crosstalk between ROS generation and the ERS-initiated UPR activation. Indeed, studies have shown that although most of the ROS in the ER is generated *via* Ero1, such redox imbalance can also be triggered by altered PERK and ATF4,functions which induce genes that facilitate elimination of ROS (Görlach et al., 2006). Furthermore, the effects of over-accumulation of ROS not only are confined to inducing oxidative stress in cardiomyocytes but also impair the antioxidant system (Liu et al., 2013), which serves as a defense system against free radicals. A thorough understanding of ROS in the pathophysiology of CVD could lead to new antioxidant-associated therapies.

Under normal physiological balance, damaging effects of free radicals are reversed by enzymatic antioxidants including superoxide dismutase (SOD), glutathione peroxidase (GPx) and catalase, and non-enzymatic antioxidants such as β-carotene, bilirubin, vitamin E, and vitamin C (He and Zuo, 2015). However, when this naïve protective system is perturbed, an excess of free radicals accumulates, overriding the antioxidant system and threatening the scavenging efficiency of existing antioxidants, thus resulting in a condition known as oxidative stress. Such alterations affect crucial cellular events such as immune cell differentiation, nervous system physiology regulation, stem cell differentiation, guanylate cyclase activation, and phosphatases, kinases, and growth factor signaling pathways (Cervantes Gracia et al., 2017).

Several studies have elucidated the effects of ROS-induced oxidative stress in CVD and emonstrated ROS as a significant contributing factor in the pathogenesis of CVD. Although ROS are well known as critical players in cardiometabolic disorders, the exact mechanisms of how ROS lead to the development of CVD is not clearly understood. Nevertheless, emerging studies have shown the role of ROS in the pathogenesis of different subtypes of CVDs including congestive heart failure, arteriosclerosis, ischemic heart disease, and xenobiotics-mediated cardiomyopathies *via* apoptosis (Ilavenil et al., 2015). Furthermore, the presence of excess ROS has oxidation-mediated damaging effects on cellular macromolecules such as lipids, cell membrane, proteins, and DNA, which alter cellular function and may contribute to the development of CVD and diabetes (Rajeshwari and Raja, 2015). Of more interest, studies have shown that in conditions of oxidative stress, nuclear E2-related factor-2 (Nrf2) transcription factor is translocated into the nucleus and binds to the antioxidant response element (ARE) through which the gene expressions of enzymatic antioxidant systems including heme oxygenase-1 (HO-1) and other enzymes involved in glutathione metabolism is regulated (He and Zuo, 2015). Thus, given the fact that apoptosis is a major causative factor for various cardiovascular pathologies, correlating apoptosis induced by ROS and ERS might work as a new perspective approach to elucidate intersecting factors for these two etiologies.

## Cardiovascular Therapeutics

### Pharmaceutical Potentials of Phytochemicals in CVD

Plants are the basis of ethnomedicines and have been used as sources of pharmaceuticals for various ailments for centuries and plants, and their derivatives have remained primary sources for lead compounds for the discovery and development of new therapeutics for several infectious and noninfectious diseases (Odey et al., 2012). Existing reports indicate that nearly 50% of all therapeutic drugs introduced by pharmaceutical industry are in some ways made from plant-based active lead compounds (de Oliveira et al., 2008). Since medicinal plants are abundant sources of phytochemicals that have wide ranges of pharmaceutical properties, the research interest and focus on plant-based bioactive compounds to search for novel medicinal agents and develop potential therapeutics for various ailments will increase dramatically in the coming decades.

Phytochemicals are bioactive compounds found naturally in plants and their products including vegetables, fruits, medicinal plants, and aromatics; and these plant chemicals are located in different anatomical parts of plants, namely, the leaves, fruits, flowers, and roots (Vasanthi et al., 2012). Phytochemicals are classified as primary and secondary metabolites. While the former serves as a primary source of energy and has roles in maintaining proper growth and development of plants, the latter play a role in plant metabolism and defense system against pathogens. Phytochemicals comprise a range of chemical groups including polyphenols, alkaloids, flavonoids, terpenoids, sterols, and vitamins, each having unique characteristics and bioactivity. Owing to their abundant phytochemical constituents, less adverse effects, and relative cost-effectiveness, plant-derived phytochemicals have been preferred over synthetic drugs (Vuddanda et al., 2010; Azwanida, 2015).

Several compounds with various potential pharmacological activities on different types of CVDs and their risk factors, including antioxidant, anti-angiogenic, anti-ischemic, anti-platelet aggregation, anti-inflammatory, anti-hypercholesterolemia, immunosuppressant, and anti-thrombotic activities, have been identified (Bradamante et al., 2004; Das and Das, 2007; Pagliaro et al., 2015). However, the underlying biomolecular mechanisms of many of these phytochemical compounds are still incomplete to substantiate their novelty and functionality. In this sense, besides the efforts and rising interests in plant-based bioactive compounds for the discovery of therapeutics for CVDs, it is also critically important to give emphasis to unfold useful information on the biological, toxicological, and structure–activity relationships. One way to achieve this is through employing a multidisciplinary systematic approach involving *in vitro*, *in vivo*, *in silico*, and molecular approaches to gain more vigorous pharmacological information.

Recent studies have revealed the role of epigenetic alterations including DNA methylation and histone modification in the progression of CVDs. Interestingly, various plant-based bioactive compounds are shown to reverse and ameliorate such epigenetic alterations. Resveratrol and curcumin (**Table 1**) are among such compounds that have been identified as potential phytochemicals that can modulate altered epigenetic signaling pathways (Schiano et al., 2015; Vietri et al., 2015). While resveratrol modulates CVS-related disorders through inhibition of phospholipase A2 and cyclooxygenase-2 (COX-2) activities and antagonizing NF-κB, TNF-α, IL-6, inducible nitric oxide synthase (iNOS), and monocyte chemoattractant protein-1 (MCP-1) (Pagliaro et al., 2015), curcumin modulates CVDs through inhibition of prostaglandin production and NF-κB activity, GRP94 induction, and modulation of the Akt/Nrf2, ERK, MAPK, p38, and JNK signaling molecules (Kapakos et al., 2012; Zhang et al., 2015; Zhang et al., 2017b). Abscisic acid (**Table 1**) has been investigated for its antidiabetes activity in obesity-related inflammation *via* peroxisome proliferator-activated receptor (PPARγ)/NF-κB signaling pathway. Carotenoids (**Table 1**) have been shown to modulate obesity-associated inflammation, atherosclerosis, and CVD through ameliorating the IGF-1, IL-1β, IL-6, and MCP-1 signals (Beukes et al., 2014). There are many more phytochemicals that have been proven to modulate risks of cardiovascular dysfunction and protect the cardiac system. Given the novelty of some of UPR-associated molecular targets, it is critically essential to emphasize the use of phytochemicals as sources of potential bioactive compounds to modulate CVDs resulting from compromised ER, epigenetics mechanisms, environmental factors, or other traditional factors. Some of the best studied phytochemicals in this context, their rich sources, their biological activities, the mechanisms of action (MOA), and associated molecular targets are described in **Table 1**.

**Table 1:** Plant-based CVD therapeutics, their pharmacological activities, mode of actions, and molecular targets.

Compound/class of phytochemical	Natural sources	Pharmacological activities	Mechanism of action (MOA)	Molecular targets	References
Resveratrol/polyphenolic compound	*Polygonum cuspidatum*, grapes, peanuts, and berries	• Inhibition of lipid peroxidation • Free radical scavenging • Inhibition of platelet aggregation • Anti-inflammatory activity • Vasorelaxation activity • Modulation of lipid metabolism	• Increases the expression of NO synthase • Decreases the expression of vasoconstrictor ET • Inhibits stress-induced *ET-1* gene expression • Alteration of eicosanoid synthesis • Attenuation of arachidonic acid and prostaglandin E2 synthesis • Inhibition of phospholipase A2 and COX-2 activities • Antagonizes NF-κB, TNF-α, IL-6, iNOS, MCP-1	AMPK, SIRT-1 pathway, NF-κB, TNF-α, IL-6, iNOS, MCP-1	(Bradamante et al., 2004; Das and Das, 2007; Pagliaro et al., 2015)
Carotenoids	*Rosa arvensis*, *Bibes nigrum*, *Fagus sylvatica*, *Malus domestica*	Obesity-associated inflammation, atherosclerosis, cardiovascular disease	……………………	IGF-1, IL-1β, IL-6, MCP-1	(Beukes et al., 2014)
*Brassica oleracea*	Broccoli, cauliflowers, Brussel sprouts, and kale	• Antioxidant • Anti-inflammatory • Antithrombotic effects.	…………………………	Nrf2, MAPK, JNK, Akt, PKB, AMPK, SIRT-1, PPARα, UCP2	(Pagliaro et al., 2015)
*Naringenin/flavonoids*	Grapefruit and orange	• Antioxidant activity • Protects LDL oxidation	• Increases the activity of antioxidant enzymes and non-enzymatic antioxidants • Inhibits generation of free radicals	LOX and COX pathways	(Vasanthi et al., 2012)
Curcumin/phenolic compound	Turmeric (*Curcuma longa*)	• Anti-inflammatory, antiplatelet, antioxidant activity	• Inhibition of prostaglandin production and NF-κB activity, an increase of cytokine production • GRP94 induction	JAK2/STAT3, AMPK/UCP2, Akt/Nrf2, ERK, MAPK p38, JNK, ICAM-1, MCP-1, and IL-8	(Kapakos et al., 2012; Zhang et al., 2015)
Isoflavones/polyphenols	Soybeans, fruits, vegetables, legumes, herbs, spices, stems, flowers	• Free radical scavenging and antioxidant activity • Anti-inflammatory activity	• Inhibition of eicosanoid generating enzymes • Modulation of the production of pro-inflammatory cytokines	ERs, Nrf1, iNO, COX-2, TNF-α, ICAM-1, VCAM-1, E-selectin, MCP-1, ERE, CVD ion channels, inhibiting calcium channels or activating potassium channels	(García-Lafuente et al., 2009) (Scholz et al., 2010)
Catechin	Green tea, apples, cocoa, and berries	• Antioxidant, anti-hypertensive, anti-inflammatory, anti-proliferative, anti-thrombogenic, hypocholesterolemia effects	• Upregulation of proinflammatory molecules by suppression of NF-κB activity • Modulation of cholesterol metabolism		(Vasanthi et al., 2012)
*Quercetin*/flavonoid	Onions, apples, and tea/	• Anti-thrombogenic • Antioxidant • Anti-platelet aggregation • Decreases superoxide production	• Inhibition of phospholipase A2 activity • Inhibition of MAP kinase activation • Decreases platelet aggregation and increases platelet-derived nitric oxide release • Inhibition of phospholipase A2 activity • Blockage of the LOX and COX pathways	LOX and COX pathways, NF-κB gene	(Vasanthi et al., 2012)
Geraniol	*Citrus* spp.	• Improve insulin resistance, suppresses HMG-CoA) reductase activity, hepatoma, melanoma, and leukemia cells	……………………	PPARγ	(Beukes et al., 2014)
*Berberine*/alkaloid	*Hydrastis canadensis*	• Anti-oxidant, anti-inflammatory • Antihyperlipidemic activity • Antihypertensive activity	• Upregulation of SOD and UCP2 • Downregulation of NADPH oxidase expression • Attenuation of ER stress-induced apoptosis • Activation of the Nrf2 pathway	JAK2/STAT3 signaling pathway, UPR signaling pathway, Nrf2 pathway, AK2/STAT3 signaling pathway, C-AMPK and MAPK/ERK pathway, NF-κB signaling pathway	(Vuddanda et al., 2010; Pagliaro et al., 2015)
*Abscisic acid*	*Rosa arvensis*, *Ribes nigrum*, *Fagus sylvatica*, *Malus domestica*	• Diabetes, obesity-related inflammation	……………………	PPARγ, NF-κB	(Beukes et al., 2014)
Sulforaphane	Broccoli, cauliflowers, Brussel sprouts, and kale	• Antioxidant activities • Anti-inflammatory activities	• Inhibition of angiogenesis • Inhibition of phase I enzymes and DNA adducts • Induction of phase II antioxidant detoxifying enzyme, • Induction of cell cycle arrest • Expression of Nrf2 • Nrf2-dependent phase 2 enzymes activation • Expression of detoxification enzymes antioxidant response elements (AREs) • Reduction of VCAM-1 synthesis	VCAM-1, Nrf2, ARE	(Pagliaro et al., 2015)

UCP2, uncoupling protein 2.

### UPR as a Potential Therapeutic Target in CVDs

The ERS and subsequent activation of the UPR have been implicated in the pathogenesis of a number of CVDs including atherosclerosis, heart failure, and ischemic heart disease (Glembotski, 2014). Targeting the UPR signaling pathway may be a potential path to modulate different types of CVDs using pharmacological interventions and to mitigate ERS-associated heart diseases.

### Therapeutic Approaches/Strategies in UPR-Mediated CVDs

There are two approaches to manipulate ERS-activated UPR or utilize the UPR components as therapeutic targets for the treatment of CVDs. These include activating selective mediators of the UPR adaptive system and deactivating the ERS-initiated proapoptotic pathway (Minamino et al., 2010). Pharmacological intervention of modulating UPR signaling components can potentially stimulate an increased capacity to alleviate protein misfolding, with positive therapeutic effects in cardiac and related metabolic diseases.

Other strategies to utilize the UPR as a target for cardiovascular therapy may include decreasing misfolded/unfolded proteins, intervening in the activation of the UPR sensors. In particular, selectively inhibiting the dissociation of GRP78 from the three ERS detectors or inhibiting the activated UPR sensors and other effectors of the UPR signaling may have efficient therapeutic potential (Liu and Dudley, 2014). While a decrease in misfolded/unfolded proteins reduces the burden caused due to abnormally deposited unfolded proteins within the lumen of ER and, as a result, leads to elimination of misfolded proteins, inhibition or attenuation of the UPR sensors or their downstream proximal or distal effectors including eIF2a/ATF4, ATF6N, and XBP1 with selective inhibitor molecules could also be a potent approach to mitigate the chronic effects of UPR activation. As illustrated in **Table 3**, apelin-13 is reported to have protective effects against ischemia/reperfusion (I/R) injury through inhibition of ERS-mediated apoptosis and the PERK/CHOP canonical UPR signaling pathway (Chen et al., 2015).

Increased expression of chaperone protein GRP78 is central to the recognition of unfolded/misfolded proteins and the coordinated activation of the UPR signaling (Toth et al., 2007). At steady state, GRP78 remains bound to the luminal domain of the three ERS sensors. However, due to its high affinity towards misfolded/unfolded proteins, GRP78 dissociates from the three sensors and binds to unfolded/misfolded proteins when ERS is induced. Clearly, targeting GRP78, which is a determinant in the initiation and progression of ERS-initiated UPR signaling process, could be a useful therapeutic approach in CVDs.

Several phytochemicals and small molecules have been investigated and identified for their modulating effect against GRP78 and downstream signaling. Bisoprolol (**Tables 2** and **3**), for example, has been reported to attenuate the UPR signaling pathway through suppression of TNF-α and IL-6 secretion, which consequently downregulates the PERK/ATF4/eIF2α signaling in myocardial I/R injury (Zhang et al., 2017b). Likewise, notoginsenoside R1 (**Table 2**) is indicated to suppress GRP78, phosphorylated PERK, ATF6, IRE1, CHOP, and phosphorylated JNK (Chen et al., 2015). There are several other compounds that have been discovered for their potential in mediating the GRP78 downstream pathways in cardiovascular-related pathologies. For example, Salubrinal93 (**Table 2**) prevents cardiac hypertrophy, one of the underlying pathophysiologies of cardiac dysfunction and hypertension, a prominent risk factor of cardiac pathologies, through dephosphorylation of eIF2α (Minamino et al., 2010). Metoprolol and propranolol (**Tables 1** and **3**), β-adrenergic receptor (β-AR) blockers, were also tested and modulated heart failure caused by cardiac hypertrophy in rats. Metoprolol and propranolol exerted this effect *via* downregulation of GRP78, XBP-1, calmodulin kinase II (CaMKII), and CHOP leading to alleviation of ERS and ERS-induced apoptosis (Chen et al., 2015).

**Table 2:** Preclinical therapeutics (natural compounds, small molecules, or peptides) targeting the UPR signaling pathway (*in vitro*).

Compound	Experimental model	Disease model	Therapeutic activity/target	Indication	References
Berberine	H9c2 cardiomyocyte	Myocardial ischemia/reperfusion (MI/R) injury	Activation of AK2/STAT3 signaling pathway and JAK2/STAT3 signaling pathway, attenuation of ERS-induced apoptosis	Downregulation of the phosphorylation levels of myocardial PERK and eIF2α, expression of ATF4 and CHOP in heart tissues	(Zhao et al., 2016)
Bisoprolol	H9c2 cardiomyocyte	Myocardial ischemia/reperfusion (I/R) injury	Inhibition of UPR signaling pathways, suppression of TNF-α and IL-6 secretion, downregulation of caspase-12 and caspase-3 expressions	Attenuation of UPR signaling pathway, downregulation of PERK/ATF4/eIF2α	(Zhang et al., 2017b)
AG1478 and 542 (small-molecule EGFR inhibitors)	H9c2 cardiomyocyte	Hyperlipidemia/obesity-induced cardiac injuries	Downregulation of TLR4/c-Src, suppression of TNFα and IL-6 expression	Reduction of cardiac inflammatory injuries and apoptosis	(Li et al., 2016)
Adiponectin U0126 (ERK1/2 inhibitor)	H9c2 cardiomyocyte	Palmitate-induced apoptosis, lipotoxicity-induced cardiomyopathy	Caspase-3 and PARP activity inhibition, ERK1/2 signaling pathway, inhibition of the Akt signaling pathway	Increase the protein level of PI3K/Akt and decrease the protein level of ERK1/2	(Wei et al., 2012)
Trigonelline	H9c2 cardiomyocyte	H_2_O_2_-induced H9c2 cell deaths, oxidative stress-mediated CVD	Downregulation of proapoptotic caspase-3 and caspase-9, upregulation of Bcl-2 and Bcl-XL expression	H_2_O_2_ induced necrosis and apoptosis	(Ilavenil et al., 2015)
Elatoside C	H9c2 cardiomyocytes	————–	Upregulation of p-STAT3 and downregulation of CHOP, GRP78, JNK	Suppression of ER stress	(Liu, H. et al., 2016)
D942 (small molecule) and curcumin (combination treatment)	Primary cardiomyocyte	Myocardial ischemia/reperfusion (I/R) injury	Activating AMPK pathway or inhibiting mTOR signaling	Upregulates autophagy and promotes cell survival	(Yang et al., 2013)
Metformin	Primary rat cardiomyocytes	N/A	Selective activation of PERK-ATF4 UPR arm, Upregulation of *CHOP* mRNA and protein	Cardioprotective effect	(Quentin et al., 2012)
Rapamycin	L6 myotubes	Diabetic cardiomyopathy (DCM)	Inhibition of mTOR (mammalian target of rapamycin), selective suppression of the IRE1–JNK signaling pathway, restoration of hyperlipidemia-induced ER stress/NF-κB-mediated pathway	Ameliorates ER stress-induced insulin resistance attenuated ER stress-induced apoptosis, ameliorates adipocyte dysfunction, upregulates autophagy	(Jung and Choi, 2016)
Notoginsenoside R1	H9c2 cardiomyocytes	—————	Suppression of GRP78, p-PERK, ATF6, IRE1, CHOP, p-JNK	—————–	(Liu, M. Q. et al., 2016)
Salubrinal93		Cardiac hypertrophy and hypertension	Prevention of eIF2a dephosphorylation		(Minamino et al., 2010)

**Table 3:** Preclinical therapeutics (natural compounds, small molecules, or peptides) targeting the UPR signaling pathway (*in vivo*).

Compound	Experimental model	Target disease	Therapeutic activity/target	Indication	References
Berberine	Male Sprague–Dawley rats	Myocardial ischemia/reperfusion (I/R) injury	Activation of AK2/STAT3 signaling pathway and JAK2/STAT3 signaling pathway, attenuation of ERS-induced apoptosis	Downregulation of the phosphorylation levels of myocardial PERK and eIF2α, expression of ATF4 and CHOP in heart tissues	(Zhao et al., 2016)
Bisoprolol	Sprague–Dawley rats	Myocardial ischemia/reperfusion (I/R) injury	Inhibition of UPR signaling pathways, suppression of TNF-α and IL-6 secretion, downregulation of caspase-12 and caspase-3 expressions	Attenuation of UPR signaling pathway, downregulation of PERK/ATF4/eIF2α	(Zhang et al., 2017b)
AG1478 and 542 (small-molecule EGFR inhibitors)	C57BL/6 mice	Hyperlipidemia/obesity-induced cardiac injuries	Downregulation of TLR4/c-Src, suppression of TNFα and IL-6 expression	Reduction of cardiac inflammatory injuries	(Li et al., 2016)
Adiponectin U0126 (ERK1/2 inhibitor)		Lipotoxicity-induced cardiomyopathy	Inhibition of the caspase-3 and PARP activity, inhibition of ERK1/2 signaling pathway, activation of the Akt signaling pathway	Increase protein level of PI3K/Akt and decrease the protein level of ERK1/2	(Wei et al., 2012)
Thioredoxin 2	Wistar rats	Diabetic cardiomyopathy	Diminish high-glucose-induced mitochondrial oxidative damage and improved ATP production		(Li et al., 2017)
D942 (small molecule) and curcumin (combination treatment)	C57BL/6 mice	Myocardial ischemia/reperfusion (I/R) injury	Activation of AMPK pathway or inhibition of mTOR signaling	Upregulates autophagy and promotes cell survival	(Yang et al., 2013)
Tauroursodeoxycholic acid (chemical chaperone)	Calreticulin-induced Transgenic mice hearts (Heart ^CRT+^)	Heart failure	Inhibition of IRE1α pathway of the UPR	Prevents cardiac fibrosis and preserve heart function	(Groenendyk et al., 2016)
Metformin (AMPK activator)	Diabetic obese mice	Cardiomyopathy	Activation of AMPK-induced PPARδ	Suppresses ER stress and protects endothelial function	(Jung and Choi, 2016)
Apelin-13	——————	Myocardial ischemia/reperfusion (I/R) injury	Modulation of PERK/CHOP, PI3K/Akt, AMPK and ERK pathways	Inhibits ERS-dependent apoptosis activation *via* the	(Lee and Ozcan, 2014)
Atorvastatin	——————	Heart failure	GRP78, caspase-12, CHOP	Inhibits ER stress-induced apoptosis	(Liu, M. Q. et al., 2016)
Metoprolol and propranolol β-adrenergic receptor (β-AR) blockers	Rats	Hypertrophic failing hearts	Downregulation of GRP78, XBP-1, and calmodulin kinase II (CaMKII), CHOP	Suppression of ERS and ERS-mediated apoptosis	(Liu, M. Q. et al., 2016)

Apoptosis of cardiomyocytes has been reported in various diseases of the cardiovascular system including myocardial ischemia, heart failure, atherosclerosis, and Diabetic cardiomyopathy (DCMP) (Lee and Gustafsson, 2009). PUMA has recently been identified as a critical proapoptotic pathway mediator and important contributor in the pathophysiology of CVDs including I/R injury. p53-up-regulated modulator of apoptosis (PUMA) is a critical contributor in ERS-induced apoptosis in cardiomyocytes, a novel proapoptotic effector upstream of Bax/Bak, and sequesters the prosurvival Bcl-2 family. This makes it an appealing therapeutic target for pharmacological intervention to attenuate myocyte apoptosis in CVD. In light of this, a study has demonstrated that ERS elicited by thapsigargin or tunicamycin led to upregulation of PUMA in cultured cardiac myocytes. Importantly, following PUMA-targeted suppression using small molecule shRNA, the cardiomyocytes managed to recover from ERS-induced cell death; ablation of PUMA has also reduced heart infarct size and attenuated apoptosis in I/R injury (Toth et al., 2007). Trigonelline (**Table 2**) is another candidate compound that is identified as having antiapoptotic effect against H_2_O_2_ induced necrosis and apoptosis in cardiomyocytes *via* inhibition of proapoptotic caspases and upregulation of the distal antiapoptotic Bcl-2 (Ilavenil et al., 2015). However, besides their antiapoptotic activity, there is a concern over some of these caspase inhibitors, as some have been reported to induce cell necrosis (Ni et al., 2016). Thus, caution is needed in targeting selected ERS markers to minimize deleterious effects.

Studies have shown that CHOP mediates the transcriptional repression and activation of proteins from Bcl-2 family (Minamino and Kitakaze, 2010). Suppression of the prosurvival protein Bcl-2 by CHOP may be a possible mechanism in CHOP-induced cellular apoptosis (Li et al., 2014). Hence, targeting this proapoptotic protein could be envisioned as a potential strategy for pharmacological intervention. Metformin is well studied for its cardioprotective effects in both cardiomyocytes and rodents. As described in **Tables 2** and **3**, metformin exerts cardioprotective effects through several mechanisms including selective activation of PERK-ATF4 UPR arm, and upregulation of CHOP mRNA and protein (Quentin et al., 2012). Berberine is a natural compound with potent modulatory effects in CVS pathologies. Berberine modulates myocardial I/R injury in rats and cell line models through activation of AK2/STAT3 and JAK2/STAT3 signaling pathways, attenuation of ERS-induced apoptosis, downregulation of the phosphorylation levels of myocardial PERK and eIF2α, and expression of ATF4 and CHOP (Zhao et al., 2016) (**Tables 2** and **3**).

The IRE1-α-dependent apoptotic signaling occurs *via* diverse pathways. Among others, the IRE1-α interacts with the adaptor protein TRAF2. IRE1α and TRAF2 interact with a mitogen-activated protein kinase ASK1, which subsequently phosphorylates JNK. Indeed, this pathway is one of the main pathways that trigger myocyte apoptosis, which may lead to various cardiovascular pathologies and chronic inflammation. Perhaps strategies to inhibit the IRE1-α/TRAF2 interaction, which would likely block the proapoptotic Ask1/JNK branch, could also be a viable therapeutic approach. Rapamycin is another compound with the potential of modulating ERS-initiated UPR activation. One way through which this compound ameliorates ER stress-induced pathologies such as insulin resistance, apoptosis, and adipocyte dysfunction, which are implicated in cardiac dysfunction, is through inhibition of mTOR (**Table 2**) (Jung and Choi 2016). In addition, rapamycin suppresses IRE-1–JNK signaling pathway, which leads to chronic inflammation and altered insulin signaling. Other studies have also demonstrated the potential of caspase inhibitors in modulating apoptosis. For example, bisoprolol is implicated in myocardial I/R injury through downregulation of caspase-12 and caspase-3, leading to downregulation of the PERK/ATF4/eIF2α UPR signaling pathway (Zhang et al., 2017b).

In general, inhibition of UPR signaling seems a promising direction in the discovery of novel therapeutics for CVDs. Many encouraging preclinical studies (*in vitro* and *in vivo*) have been conducted, but gaps and limitations remain in understanding many underpinning scenarios. For example, it is clearly understood that under normal circumstances, activation of the UPR has a protective or adaptive role, but chronic activation will have deleterious effects. Unlike acute interventions, which could be undertaken with acceptable risks of side effects, Kim et al. (2008) have discussed in their review that prolonged perturbation of the ERS-initiated UPR signaling in a broad systemic manner could potentially have indiscriminate deleterious effects on some tissues and organs. This necessitates the need for a critical time point and temporary therapeutic strategies in targeting the UPR for therapeutic intervention. Perhaps a specific approach to assess which of the three ERS signaling arms and associated mediators are activated in response to ERS-inducing stimuli would be very helpful to develop a better strategy for UPR targeted therapeutics. According to Ni et al. (2016) TNF-α targeted inhibition of caspases using inhibitor molecule, ZVAD-fmk, has shown an inhibitory effect against apoptosis at early intervention but promoted cell necrosis in prolonged treatment (Ni et al., 2016). This effect has been observed in both *in vitro* and *in vivo* models, raising concern over the safety of emerging caspase inhibitors, which are undergoing clinical trials. As such, caution is needed in targeting selected ERS markers to minimize deleterious effects. This necessitates the need for further investigation to elucidate how and when cells decide to enter necrotic or apoptotic pathways as well as the cross-talk between different cell death mechanisms.

#### Therapeutics Under Clinical Trials Targeting ERS and UPR

The past decade has seen fast developments in therapeutic approaches to treat various metabolic disorders including high-lipid-induced insulin resistance and T2DM. Some of the potential agents targeting ER pathway could possibly evolve as effective strategies for obesity-associated complications. Tauroursodeoxycholic acid (TUDCA) and sodium phenylbutyrate (PBA) (**Table 4**) are some of the drugs that have been investigated for their therapeutic effects in obese people who have high-lipid-associated insulin resistance. The pharmacological effects and the mechanism of action (MOA) of these drugs have been linked to a decrease in ERS (NCT00771901 and NCT02344186). According to the investigation, TUDCA and PBA modulate obesity-related insulin resistance by reverting ERS and associated biomarkers, thus improving insulin action (NCT00771901). Liraglutide (**Table 4**) is another pharmacological drug that was found to ameliorate ER stress and adipose tissue in obese people who have T2DM (NCT02344186). Alpha-lipoic acid (ALA) (**Table 4**) is a natural compound that is a potent antioxidant and free radical scavenger with attractive pharmacological activity for the treatment of T2DM. This compound has undergone phase 4 clinical trials, and its pharmacological effects have been linked to modulation of the insulin signal pathways and antagonizing the oxidative and inflammatory stresses (NCT01056497). All these findings implicate the significance of targeting ERS and associated pathways for pharmacological intervention of common metabolic disorders including insulin resistance, T2DM, and CVD.

**Table 4:** Clinical trials of therapeutics targeting ERS and UPR.

Compound	Disease	Targeted mechanisms	Clinical trial phase	Reference
**Tauroursodeoxycholic acid (TUDCA)**	Insulin resistance	ERS	N/A	*NCT00771901*
**Sodium phenylbutyrate (PBA)**	Insulin resistance	ERS	N/A	*NCT00771901*
**Liraglutide**	Type 2 diabetes	ERS	Phase 4	*NCT02344186*
**Buphenyl**	Diabetes Insulin resistance	ERS	Phase 4	*NCT00533559*
**Alpha-lipoic acid (ALA)**	Type 2 diabetes Prediabetes	ERS	Phase-4	*NCT01056497*

### Challenges and Future Perspectives

As discussed earlier, ERS and associated cellular events, mainly ERS-induced myocyte apoptosis, have been implicated in the initiation and progression of different types of CVDs. In line with this, alleviation of ERS and its constituents has recently emerged as a potential therapeutic target to intervene in CVDs such as heart failure, hypertension, and atherosclerosis (Zhang et al., 2017b). Given that myocyte apoptosis is one of the major contributors to CVDs, perhaps pathways involved in this event offer an attractive therapeutic target to combat the diseases. In their review, Toth et al. highlighted that the ERS pathway and mediating molecules that govern cell survival and cell death are the best targets for pharmacological intervention. However, there are multiple downstream pathways that lead to proapoptotic events in ERS; as a result, therapeutic strategies targeting or modulating a single apoptotic pathway may not be enough to preserve the survival of cardiomyocytes (Xu et al., 2005; Kim et al., 2008). A deep understanding of the interconnecting pathways involved in ER-initiated cell death is essential to fully validate targets for drug discovery.

Most of the components of UPR signaling, including the proapoptotic pathway, are widely expressed; thus, blocking their activity may trigger unpredictable adverse effects. This is particularly true in conditions where delivery of the ligand to the target cell types or tissue fails. Hence, further research in ERS is needed to elucidate the best therapeutic targets within the various interconnecting networks. Moreover, molecular mechanisms of ERS-linked proteins and genes regulation involved in the initiation and progression of ERS-induced cell death are essential for identification of novel molecular markers for future drug discovery.

## Concluding Remarks

CVDs constitute a major challenge and are at an epidemic proportion worldwide. Despite significant progress made thus far, the development of novel and effective therapeutics for CVDs has continued to be a major research goal due to limitations and reported side effects in existing therapies. The impact of ERS-induced activation of UPR signaling in the pathophysiology of CVDs is well documented in numerous *in vitro* and animal studies. The apoptotic event that is triggered following chronic ERS has been implicated in the development and progression of CVDs. Nevertheless, further research is warranted to identify the best drug targets within the ERS pathway to expedite the fight against cardiovascular and other metabolic diseases.

ERS pathways may offer attractive targets to combat cardiovascular pathologies and discover therapeutic alternatives in disease processes. However, there are still many constraints in terms of understanding the molecular mechanisms of this phenomenon. As such, understanding the underlying molecular mechanisms of events including the stress-associated UPR activation and ERS-induced apoptosis resulting from protracted ERS is of paramount importance. This will lead to gain of substantial biomolecular information, which will help in identifying novel targets for the discovery of new therapeutic approaches and the development of effective drugs. Therefore, a better and improved understanding of the molecular mechanisms underpinning activation of UPR and the ensuing cellular apoptosis in CVD will provide us with new and novel biological targets that can be utilized for potential therapeutic interventions and drug discovery for CVDs.

Meanwhile, owing to their immense health benefits, fewer side effects, and a broad spectrum of pharmacological potentials against different ailments, phytochemicals have become the primary focus in the discovery and development of therapeutics for a range of diseases including CVD. As described in **Tables 1**, **2**, and **3**, several promising compounds that modulate CVDs and protect CVS by interfering with ERS and UPR signaling and associated mediator molecules have been identified. Thus, given the significant role of phytochemicals as potential bioactive compounds to modulate CVDs resulting from compromises in epigenetics mechanisms or other traditional risk factors, it is a better alternative and perhaps a promising avenue to emphasize the research on such and other bioactive compounds with multidisciplinary approaches to foster the ongoing efforts to develop novel drugs and better therapeutic approaches for CVDs.

## Author Contributions

OMA contributed to the literature review, drafted the manuscript, and critically revised and amended the manuscript. AA did the supervision. All authors revised and approved the final version of the manuscript.

## Funding

This review is supported by Taylor’s Research Grant—Major Funding Scheme (TRGS-MFS) Project No. TRGS/MFS/2/2016/SOM/003.

## Conflict of Interest Statement

The authors declare that the research was conducted in the absence of any commercial or financial relationships that could be construed as a potential conflict of interest.

## Abbreviations

ACE, acetylcholine enzyme; ATF4, activating transcription factor 4; ATF6, activating transcription factor 6; ATP, adenosine triphosphate; APN, adiponectin; ATMs, adipose tissue macrophages; ARE, antioxidant response element; BiP, binding immunoglobulin protein; CVD, cardiovascular dysfunction; CVS, cardiovascular system; CHOP, C homologous protein; c-JNK, C-Jun N-terminal kinase; CD36, a cluster of differentiation 36; CAD, coronary artery disease; CRP, C-reactive protein; COX-2, cyclooxygenase-2; DAMPs, danger-associated molecular patterns; DNA, deoxyribonucleic acid; DCM, diabetic cardiomyopathy; DAGs, diacylglycerols; ER, endoplasmic reticulum; ERAD, endoplasmic reticulum-associated degradation; ERS, endoplasmic reticulum stress; ERSE, endoplasmic reticulum stress response element; eNOS, endothelial nitric oxide synthase; FAT, fatty acid translocase; ECG, echocardiography; ERK, an extracellular signal-regulated kinase; FGF, fibroblast growth factor; FFA, free fatty acids; GRP78, glucose-regulated protein 78; GRP94, glucose-regulated protein 94; GLUT1, glucose transporter 1; GLUT4, glucose transporter 4; GPx, glutathione peroxidase; GADD34, growth arrest and DNA-damage-inducible protein 34; HbA1c, glycosylated hemoglobin; HO-1, heme oxygenase-1; H2O2, hydrogen peroxide; HO, hydroxyl radicals; eIF2a, initiation factor 2a; IRE1, inositol-requiring enzyme 1; IRS-1, insulin receptor substrate 1; IRS-2, insulin receptor substrate 2; IL-6, interleukin-6; IMT, intima-media thickness; ICAM-1, intracellular adhesion molecule 1; LPS, left ventricular dysfunction; LVD, lipopolysaccharide; LDL, low-density lipoprotein; LO, lypoxidases; NOX, mitochondrial NADPH oxidases; MPO, myeloperoxidases; NO, nitric oxide; ATF6N, N-terminus of ATF6a; Nrf2, nuclear E2-related factor-2; NF-κB, nuclear factor-kappa B; NOD, nucleotide oligomerization domain; OxPL, oxidized phospholipids; PAMPs, pathogen-associated molecular patterns; PRRs, pattern-recognition receptors; OONO−, peroxynitrite; PERK, PKR-like ER kinase; PDI, protein disulfide isomerase; ROS, reactive oxygen species; RAGE, receptor for advanced glycation end-products; O2, singlet oxygen; S1Ps, site one proteases; S2Ps, site two proteases; sXBP1, spliced X-box binding protein 1; SAT, subcutaneous adipose tissue; −O2, superoxide; SOD, superoxide dismutase; TAGs, triacylglycerides; TLRs, Toll-like receptors; TNF-α, tumor necrosis factor-α; TRAF2, tumor necrosis factor-α receptor-associated factor 2; T2DM, type-2 diabetes mellitus; VCAM-1, vascular cell adhesion molecule 1; LVH, ventricular hypertrophy; VLDL, very-low-density lipoprotein; VAT, visceral adipose tissue; WHO, World Health Organization; XO, xanthine oxidases.
