# Supplementary material for: Endoplasmic Reticulum Stress Activates Unfolded Protein Response Signaling and Mediates Inflammation, Obesity, and Cardiac Dysfunction: Therapeutic and Molecular Approach
Source: Front Pharmacol. 2019 Sep 10;10:977. doi: 10.3389/fphar.2019.00977 (PMC6747043; doi:10.3389/fphar.2019.00977)
Supplement: Supplementary file 1 [file DataSheet_1.docx]

Supplementary Material


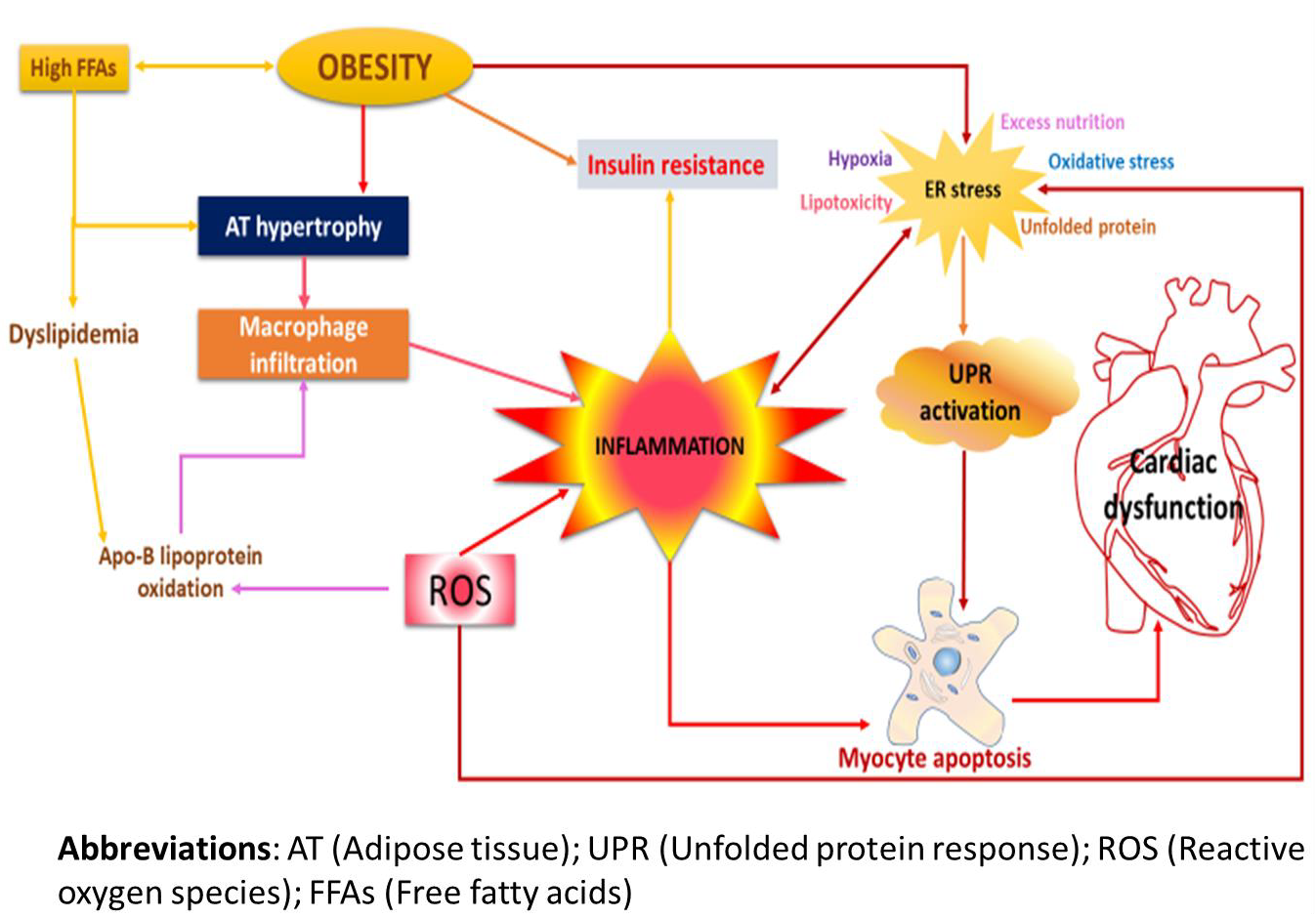


**Graphical abstract.** Correlation between excess nutrition/overweight/obesity and other risk factors such as hyperlipidemia, FFAs, ROS, ERS, inflammation and insulin resistance in the pathophysiology of obesity-associated cardiac dysfunction.
